# Cyanobacteria of Greece: an annotated checklist

**DOI:** 10.3897/BDJ.4.e10084

**Published:** 2016-11-01

**Authors:** Spyros Gkelis, Iordanis Ourailidis, Manthos Panou, Nikos Pappas

**Affiliations:** ‡ Department of Botany, School of Biology, Aristotle University of Thessaloniki, Thessaloniki, Greece

**Keywords:** blue-green algae, Cyanophyceae, bacteria, microorganisms, Aegean Sea, freshwater, marine, checklist, biodiversity, Mediterranean

## Abstract

**Background:**

The checklist of Greek Cyanobacteria was created in the framework of the Greek Taxon Information System (GTIS), an initiative of the LifeWatchGreece Research Infrastructure (ESFRI) that has resumed efforts to compile a complete checklist of species reported from Greece. This list was created from exhaustive search of the scientific literature of the last 60 years. All records of taxa known to occur in Greece were taxonomically updated.

**New information:**

The checklist of Greek Cyanobacteria comprises 543 species, classified in 130 genera, 41 families, and 8 orders. The orders Synechococcales and Oscillatoriales have the highest number of species (158 and 153 species, respectively), whereas these two orders along with Nostocales and Chroococcales cover 93% of the known Greek cyanobacteria species. It is worth mentioning that 18 species have been initially described from Greek habitats. The marine epilithic *Ammatoidea aegea* described from Saronikos Gulf is considered endemic to this area. Our bibliographic review shows that Greece hosts a high diversity of cyanobacteria, suggesting that the Mediterranean area is also a hot spot for microbes.

## Introduction

The history of natural science is directly linked to Greek civilization, since the older written references to plants, animals, and algae are found in the Homeric Epics dating back to the 8^th^ century BC; the first record of algae in the Western literature appears in Homer’s Iliad: “the dark waves uprear their heads and scatter their sea-wrack (*phycos*) in all directions” [*Iliad* IX.1 translated by Batler (1998)]. The Greek philosopher, and first marine biologist, Aristotle, who has established scientific knowledge for many animal and plant taxa (see Voultsiadou 2007) reported “red snow” in 4^th^ century BC: “after lying for some time, snow turns slightly red” (*Historia Animalium* 552b8 translated by Peck 1970); this is considered as one of the oldest records of snow algae [unicellular Chlorophyta, like *Chlamydomonas* or *Chloromonas* according to Duval et al. (1999)].

In modern times, research on Greek microflora, Cyanobacteria included, started in the 20^th^ century with the studies of freshwater algae by Stanković (1931) and Skuja (1937). In the subsequent two decades, no published work in microflora can be traced until the limnological survey by Ananiadis (1956). During the 1960s Konstantinos Anagnostidis performs two extensive studies of Cyanobacteria from thermal springs (Anagnostidis 1961) and sulphur-communities (Sulphuretum) in marine and freshwater habitats of Greece (Anagnostidis 1968). These taxonomic studies revealed the occurrence of a great number of cyanobacteria taxa, led to the description of several new species (e.g. *Geitlerinema apolloniae* Anagnostidis, 2001; *Leptolyngbya phormidioides* (Anagnostidis) Anagnostidis & Komárek, 1988; *Pseudanabaena lonchoides* Anagnostidis, 1961; *Pseudophormidium battersii* (Gomont) Anagnostidis, 2001; *Borzia periklei* Anagnostidis in (Anagnostidis and Komárek 1988)), and were later widely used to establish the modern classification system of Cyanobacteria (Anagnostidis and Komárek 1985, Anagnostidis and Komárek 1988, Anagnostidis and Komárek 1990, Komárek and Anagnostidis 1986, Komárek and Anagnostidis 1989). Although a lot of the valuable taxonomic, ecological, and biogeographical information recorded in Anagnostidis' monographs is now contained in the “Süsswasserflora von Mitteleuropa” series (Komárek 2013, Komárek and Anagnostidis 1999, Komárek and Anagnostidis 2005, Komárek 2013), many details of his work remain unavailable to the broader scientific community since they were published in Greek.

The systematic research on Greek Cyanobacteria started in the 1980s with a series of publications on cyanobacterial taxonomy and species composition (Anagnostidis and Economou-Amilli 1978, Anagnostidis and Economou–Amilli 1980, Anagnostidis et al. 1981, Anagnostidis et al. 1982, Overbeck et al. 1982, Anagnostidis et al. 1983, Anagnostidis and Pantazidou 1985, Anagnostidis and Pantazidou 1988a, Anagnostidis and Pantazidou 1988b, Anagnostidis and Pantazidou 1988c, Hindak and Moustaka 1988, Anagnostidis et al. 1988a, Anagnostidis and Pantazidou 1991a, Anagnostidis and Pantazidou 1991b, Anagnostidis and Roussomoustakaki 1991, Tryfon 1996), as well as on population dynamics (Moustaka-Gouni 1988, Moustaka-Gouni and Nikolaidis 1990, Moustaka-Gouni and Nikolaidis 1992, Tafas and Economou-Amilli 1997, Tryfon et al. 1997, Montesanto et al. 1999, Moustaka-Gouni et al. 2000, Temponeras et al. 2000, Vardaka et al. 2000) regarding both the freshwater and marine waterbodies. The recent research on cyanobacterial diversity in Greece comprises polyphasic or multidisciplinary studies of cyanobacteria strains (Gkelis et al. 2005b, Lamprinou et al. 2011, Lamprinou et al. 2013a, Lamprinou et al. 2013b, Gkelis et al. 2015b, Gkelis and Panou 2016, Bravakos et al. 2016), natural populations (Moustaka-Gouni et al. 2009, Moustaka-Gouni et al. 2010, Papadimitriou et al. 2013, Gkelis and Zaoutsos 2014, Gkelis et al. 2014, Gkelis et al. 2015a), as well as ecological works (e.g. Lamprinou et al. 2009, Katsiapi et al. 2011, Katsiapi et al. 2012).

Despite the increased research efforts, knowledge of diversity and distribution of Cyanobacteria in Greece is still inconsistent since no list on local cyanobacterial flora has ever been published. Modern biodiversity research faces an ever increasing rate of data generation and efforts are made to structure, aggregate, link and process these data in a meaningful way (Koureas et al. 2016). Currently, the Greek Taxon Information System (GTIS), an initiative launched by the LifeWatchGreece Research Infrastructure (ESFRI), is resuming efforts to compile a database of all species reported from Greece; a prerequisite for GTIS is to have checklists for each taxonomic group (Bailly et al. 2016). The aim of this study is to create a checklist of cyanobacterial taxa inhabiting the area of Greece on the basis of the recent taxonomic revisions.

## Materials and methods

The Checklist of Greek Cyanobacteria was created in the framework of the Greek Taxon Information System (GTIS), an initiative of the LifeWatchGreece Research Infrastructure (ESFRI) that has resumed efforts to compile a complete checklist of all species reported from Greece (Bailly et al. 2016). In that publication, a methodology is described to produce Preliminary Checklists only. However, in the present case of Cyanobacteria, the status of the list for Greece was quite advanced, and the recent primary literature was exhaustively searched for this work; the present list is thus considered as an updated, annotated, and archived checklist.

The data for this study were collected through extensive search in the scientific literature of the last 60 years. The search was performed in Scopus and Web of Science using the keywords: Greece AND cyanobacteria, Greece AND phytoplankton, Greece AND cyanoprokaryota, Greece AND blue-green algae. Furthermore, the two exhaustive monographs by Anagnostidis (Anagnostidis 1961, Anagnostidis 1968) were studied and the cyanobacterial taxa were recorded. All geographical areas of Greece and all biomes (freshwater, marine, terrestrial, caves etc.) and biotic forms (planktic, benthic, periphytic, endolithic, epizoic etc.) were considered.

The taxonomic status of the recorded cyanobacterial taxa was checked and updated, where necessary, to the currently accepted taxonomically species using Komárek and Anagnostidis (1999), Komárek and Anagnostidis (2005), Komárek (2013), and the AlgaeBase (Guiry and Guiry 2016). The species were classified to suprageneric taxa according to the latest taxonomic revisions on the basis of polyphasic methods (Komárek et al. 2014, Komárek 2016). Not valid species, species of unclear taxonomic status, and species transferred to other phyla were excluded from the list. Also, infraspecific taxa that are not valid or need revision were not included in the list. The first publication mentioning a taxon's occurrence in Greece and the original name used in that record is given under Notes and Nomeclature, respectively.

## Data resources

The data collected were published in Global Biodiversity Information Facility (GBIF) through the Integrated Publishers Toolkit (IPT) according to the Darwin Core Archive biodiversity informatics data standard and are made publicly available through GBIF (UUID: 655027fc-76ea-447a-a443-f36dd2e853d7, DOI: 10.15468/lkj0mr).

## Checklists

### List of Cyanobacteria known to occur in Greece

#### Ammatoidea aegaea

Anagnostidis & Pantazidou, 1991

Ammatoidea aegea

##### Notes

Anagnostidis and Pantazidou 1991b

#### Anabaena cylindrica

Lemmermann, 1896

Anabaena cf. cylindrica

##### Notes

Gkelis et al. 2015b

#### Anabaena inaequalis

Bornet & Flahault, 1888

Anabaena inaequalis

##### Notes

Tafas and Economou-Amilli 1997

#### Anabaena laxa

A. Braun in Bornet & Falhault, 1888

Anabaena laxa

##### Notes

Anagnostidis 1961

#### Anabaena oscillarioides

Bory ex Bornet & Flahault, 1888

Anabaena oscillarioides

##### Notes

Anagnostidis 1961

#### Anabaena perturbata

H. Hill, 1976

Anabaena pertubata

##### Notes

Moustaka 1988

#### Anabaena planctonica

Brunnthaler, 1903

Anabaena solitaria f. planctonica

##### Notes

Moustaka 1988

#### Anabaena sphaerica

Bornet & Flahault, 1888

Anabaena sphaerica

##### Notes

Tafas and Economou-Amilli 1997

#### Anabaena torulosa

Lagerheim ex Bornet & Flahault, 1888

Anabaena torulosa

##### Notes

Anagnostidis 1961​

#### Anabaenopsis arnoldii

Aptekar, 1926

Anabaenopsis arnoldii

##### Notes

Moustaka-Gouni et al. 2007

#### Anabaenopsis circularis

(G. S. West) Woloszynska & V.Miller, 1923

Anabaenopsis cf. elenkinii f. circularis

##### Notes

Moustaka 1988​

#### Anabaenopsis circularis

(G. S. West) Woloszynska & V.Miller, 1923

Anabaenopsis cunningtonii

##### Notes

Vardaka et al. 2005

#### Anabaenopsis elenkinii

V. V. Miller, 1923

Anabaenopsis elenkinii

##### Notes

Vardaka et al. 2005​

#### Anabaenopsis milleri

Woronichin, 1929

Anabaenopsis milleri

##### Notes

Lanaras et al. 1989

#### Anabaenopsis tanganyikae

(G. S. West) Woloszynska & V.Miller, 1923

Anabaenopsis tanganyikae

##### Notes

Hindak and Moustaka 1988

#### Anathece clathrata

(W. & G. S. West) Komárek, Kastovsky & Jezberová, 2011

Aphanothece clathrata

##### Notes

Anagnostidis 1968​

#### Anathece endophytica

(W. & G. S. West) Komárek, Kastovsky & Jezberová, 2011

Aphanothece nidulans var. endophytica

##### Notes

Hindak and Moustaka 1988

#### Anathece minutissima

(West) Komárek, Kastovsky & Jezberová, 2011

Aphanothece minutissima

##### Notes

Tryfon et al. 1997

#### Aphanizomenon favaloroi

S.H.Otaño, 2012

Aphanizomenon favaloroi

##### Notes

Moustaka-Gouni et al. 2016

#### Aphanizomenon flos-aquae

Ralfs ex Bornet & Flahault, 1886

Aphanizomenon flos-aquae

##### Notes

Ananiadis 1956

#### Aphanizomenon gracile

Lemmermann, 1907

Aphanizomenon gracile

##### Notes

Anagnostidis 1968​

#### Aphanocapsa biformis

A. Braun in P. Richter, 1879

Aphanocapsa biformis

##### Notes

Anagnostidis 1961​

#### Aphanocapsa holsatica

(Lemmermann) G. Cronberg & Komárek, 1994

Microcystis cf. holsatica

##### Notes

Hindak and Moustaka 1988

#### Aphanocapsa conferta

(W. & G. S. West) Komárková-Legnerová & Cronberg, 1994

Aphanocapsa elachista var. conferta

##### Notes

Tryfon et al. 1997

#### Aphanocapsa delicatissima

W. & G. S. West, 1912

Aphanocapsa delicatissima

##### Notes

Moustaka-Gouni 1988

#### Aphanocapsa elachista

W. & G. S. West, 1912

Aphanocapsa elachista

##### Notes

Anagnostidis 1968​

#### Aphanocapsa fusco-lutea

Hansgirg, 1892

Aphanocapsa fusco-lutea

##### Notes

Lamprinou et al. 2012

#### Aphanocapsa grevillei

(Berkeley) Rabenhorst, 1865

Aphanocapsa grevillei

##### Notes

Anagnostidis 1961​

#### Aphanocapsa incerta

(Lemmermann) G. Cronberg & Komárek, 1994

Aphanocapsa incerta

##### Notes

Tryfon et al. 1996

#### Aphanocapsa marina

Hansgirg, 1892

Aphanocapsa marina

##### Notes

Anagnostidis 1968​

#### Aphanocapsa muscicola

(Meneghini) Wille, 1919

Aphanocapsa muscicola

##### Notes

Anagnostidis 1961​

#### Aphanocapsa parietina

(Nägeli ex Kützing) Nägeli, 1849

Aphanocapsa parientina

##### Notes

Lamprinou et al. 2012​

#### Aphanocapsa raspaigellae

(Hauck) Frémy in Feldmann, 1933

Aphanocapsa raspaigellae

##### Notes

Anagnostidis 1968​

#### Aphanocapsa rivularis

(Carmichael) Rabenhorst, 1865

Aphanocapsa anodontae

##### Notes

Anagnostidis 1968​

#### Aphanocapsa salina

Woronichin (Voronichin), 1929

Aphanocapsa salina

##### Notes

Anagnostidis and Golubic 1966

#### Aphanocapsa sesciacensis

Frémy, 1928

Aphanocapsa sescianensis

##### Notes

Anagnostidis and Golubic 1966

#### Aphanocapsa thermalis

Brügger, 1863

Aphanocapsa thermalis

##### Notes

Anagnostidis 1961​

#### Aphanothece castagnei

(Kützing) Rabenhorst, 1865

Aphanothece castagnei

##### Notes

Anagnostidis 1961​

#### Aphanothece rubra

Liebetanz, 1926

Aphanothece cf. rubra

##### Notes

Lamprinou et al. 2012​

#### Aphanothece rufescens

Hansgrig, 1892

Aphanothece cf. rufescens

##### Notes

Lamprinou et al. 2012​

#### Aphanothece salina

Elenkin & Danilov, 1915

Aphanothece cf. salina

##### Notes

Anagnostidis and Roussomoustakaki 1991

#### Aphanothece marina

(Ercegovic) Komárek & Anagnostidis, 1995

Synechococcus marinus

##### Notes

Anagnostidis 1968​

#### Aphanothece microscopica

Nägeli, 1849

Aphanothece microscopica

##### Notes

Anagnostidis 1968​

#### Aphanothece microspora

(Meneghini) Rabenhorst, 1863

Aphanothece microspora

##### Notes

Anagnostidis 1968​

#### Aphanothece nidulans

Richter in Wittrock & Nordstedt, 1884

Aphanothece nidulans

##### Notes

Anagnostidis 1961​

#### Aphanothece pallida

(Kützing) Rabenhorst, 1863

Aphanothece pallida

##### Notes

Lamprinou et al. 2012​

#### Aphanothece saxicola

Nägeli, 1849

Aphanothece saxicola

##### Notes

Anagnostidis 1968​

#### Aphanothece stagnina

(Sprengel) A. Braun in Rabenhorst, 1863

Aphanothece stagnina

##### Notes

Anagnostidis 1968​

#### Arthrospira fusiformis

(Voronikhin) Komárek & J. W. G. Lund, 1990

Arthrospira fusiformis

##### Notes

Moustaka-Gouni et al. 2007

#### Arthrospira platensis

Gomont, 1892

Arthrospira platensis

##### Notes

Vardaka et al. 2005

#### Asterocapsa aerophytica

F. Lederer, 2000

Asterocapsa aerophytica

##### Notes

Lamprinou et al. 2012​

#### Asterocapsa divina

Komárek, 1993

Asterocapsa divina

##### Notes

Lamprinou et al. 2012​

#### Asterocapsa jilinica

H. X. Xiao, 2000

Asterocapsa jilinica

##### Notes

Lamprinou et al. 2012​

#### Asterocapsa sinica

Liang & Chen, 1985

Asterocapsa sinica

##### Notes

Lamprinou et al. 2012​

#### Blennothrix brebissonii

(Kützing ex Gomont) Anagnostidis & Komárek, 1988

Hydrocoleum brebissonii

##### Notes

Anagnostidis 1968​

#### Blennothrix glutinosa

(Gomont ex Gomont) Anagnostidis & Komárek, 2001

Hydrocoleum glutinosum

##### Notes

Anagnostidis 1968

#### Blennothrix heterotricha

(Gomont ex Gomont) Anagnostidis & Komárek, 1988

Hydrocoleum heterotrichum

##### Notes

Anagnostidis 1968​

#### Blennothrix lyngbyacea

(Kützing ex Gomont) Anagnostidis & Komárek, 1988

Hydrocoleum lyngbyaceum

##### Notes

Anagnostidis and Golubic 1966

#### Borzia periklei

Anagnostidis in Anagnostidis & Komárek, 1988

Borzia trilocularis

##### Notes

Anagnostidis 1977

#### Borzia starkii

Schiller, 1954

Borzia starkii

##### Notes

Anagnostidis 1977

#### Borzia susedana

Ercegovic, 1925

Borzia susedana

##### Notes

Anagnostidis 1977

#### Borzia trilocularis

Cohn ex Gomont, 1892

Borzia trilocularis

##### Notes

Anagnostidis 1968​

#### Brachytrichia quoyi

Bornet & Flahault, 1886

Brachytrichia quiyi

##### Notes

Anagnostidis 1968​

#### Calothrix aeruginea

Thuret ex Bornet & Flahault, 1886

Calothrix aeruginea

##### Notes

Anagnostidis 1968

#### Calothrix braunii

Bornet & Flahault, 1886

Calothrix braunii

##### Notes

Anagnostidis 1968​

#### Calothrix fusca

Bornet & Flahault, 1886

Calothrix cf. fusca

##### Notes

Lamprinou et al. 2012​

#### Calothrix confervicola

C. Agardh ex Bornet & Flahault, 1886

Calothrix confervicola

##### Notes

Anagnostidis 1968​

#### Calothrix contarenii

Bornet & Flahault, 1886

Calothrix contarenii

##### Notes

Anagnostidis 1968​

#### Calothrix javanica

De Wildeman, 1897

Calothrix javanica

##### Notes

Anagnostidis et al. 1981

#### Calothrix marchica

Lemmermann, 1914

Calothrix marchica

##### Notes

Anagnostidis et al. 1981

#### Calothrix parietina

Thuret ex Bornet & Flahault, 1886

Calothrix parietina

##### Notes

Anagnostidis 1968​

#### Calothrix pulvinata

C. Agardh ex Bornet & Flahault, 1886

Calothrix pulvinata

##### Notes

Anagnostidis and Golubic 1966

#### Calothrix scopulorum

C. Agardh ex Bornet & Flahault, 1886

Calothrix scopulorum

##### Notes

Anagnostidis and Golubic 1966

#### Calothrix stagnalis

Gomont, 1895

Calothrix stagnalis

##### Notes

Anagnostidis et al. 1981

#### Calothrix thermalis

Hasngirg ex Bornet & Flahault, 1886

Calothrix thermalis

##### Notes

Anagnostidis 1968​

#### Chamaesiphon confervicola

A. Braun in Rabenhorst, 1865

Chamaesiphon curvatus

##### Notes

Anagnostidis 1968​

#### Chamaesiphon geitleri

A. Braun in Rabenhorst, 1865

Chamaesiphon geitleri

##### Notes

Anagnostidis 1968

#### Chamaesiphon incrustans

Grunow in Rabenhorst, 1865

Chamaesiphon incrustans

##### Notes

Anagnostidis 1968​

#### Chamaesiphon polonicus

(Rostafinski) Hansgirg, 1893

Chamaesiphon polonicus

##### Notes

Anagnostidis 1968

#### Chamaesiphon polymorphus

Geitler, 1925

Chamaesiphon polymorphus

##### Notes

Anagnostidis 1968​

#### Chlorogloea microcystoides

Geitler, 1926

Chlorogloea microcystoides

##### Notes

Anagnostidis 1961​

#### Chlorogloea novacekii

Komárek & Montejano, 1994

Chlorogloea novacekii

##### Notes

Lamprinou et al. 2012​

#### Chlorogloea rivularis

(Hansgirg) Komárek & Anagnostidis, 1995

Xenococcus rivularis

##### Notes

Anagnostidis 1968​

#### Chroococcidiopsis doonensis

R. B. Singh, 1968

Chroococcidiopsis doonensis

##### Notes

Lamprinou et al. 2009

#### Chroococcidiopsis kashayi

Friedmann, 1961

Chroococcidiopsis kashaii

##### Notes

Lamprinou et al. 2012​

#### Chroococcidiopsis thermalis

Geitler, 1933

Chroococcidiopsis thermalis

##### Notes

Anagnostidis and Pantazidou 1988a

#### Chroococcidium gelatinosum

Geitler, 1933

Chroococcidium gelatinosum

##### Notes

Metaxatos et al. 2003

#### Chroococcus aphanocapsoides

Skuja ex Joosten, 2006

Chroococcus cf. aphanocapsoides

##### Notes

Lamprinou et al. 2012​

#### Chroococcus subsphaericus

N. L. Gardner, 1927

Chroococcus cf. subsphaericus

##### Notes

Lamprinou et al. 2012​

#### Chroococcus cohaerens

(Brébisson) Nägeli, 1849

Chroococcus cohaerens

##### Notes

Lamprinou et al. 2009

#### Chroococcus dispersus

(Keissler) Lemmermann, 1904

Chroococcus dispersus

##### Notes

Anagnostidis 1968

#### Chroococcus helveticus

Nägeli, 1849

Chroococcus helveticus

##### Notes

Anagnostidis et al. 1981

#### Chroococcus lithophilus

Ercegovic, 1925

Chroococcus lithophilus

##### Notes

Lamprinou et al. 2012​

#### Chroococcus microscopicus

Komárková-Legnerová & G. Cronberg, 1994

Chroococcus microscopicus

##### Notes

Tryfon et al. 1997

#### Chroococcus minor

(Kützing) Nägeli, 1849

Chroococcus minor

##### Notes

Anagnostidis 1961​

#### Chroococcus minutus

(Kützing) Nägeli, 1849

Chroococcus minutus

##### Notes

Anagnostidis 1961​

#### Chroococcus spelaeus

Ercegovic, 1925

Chroococcus spelaeus

##### Notes

Lamprinou et al. 2009

#### Chroococcus subnudus

(Hansgirg) G. Cronberg & J. Komárek, 1994

Chroococcus subnudus

##### Notes

Lamprinou et al. 2012​

#### Chroococcus tenax

(Kirchner) Hieronymus, 1892

Chroococcus tenax

##### Notes

Lamprinou et al. 2009

#### Chroococcus thermalis

(Meneghini) Nägeli, 1849

Chroococcus thermalis

##### Notes

Radea et al. 2010

#### Chroococcus turgidus

(Kützing) Nägeli, 1849

Chroococcus turgidus

##### Notes

Anagnostidis 1961​

#### Chroococcus turicensis

(Nägeli) Hansgirg, 1887

Chroococcus turicensis

##### Notes

Lamprinou et al. 2012​

#### Chroococcus varius

A. Braun in Rabenhorst, 1876

Chroococcus varius

##### Notes

Anagnostidis 1968​

#### Chroococcus westii

Boye-Petersen, 1923

Chroococcus westii

##### Notes

Lamprinou et al. 2012​

#### Chrysosporum bergii

(Ostenfeld) E. Zapomelová, O. Skácelová, P. Pumann, R. Kopp & E. Janecek, 2012

Anabaena bergii

##### Notes

Katsiapi et al. 2011

#### Chrysosporum ovalisporum

(Forti) E. Zapomelová, O. Skácelová, P. Pumann, R. Kopp & E. Janecek, 2012

Aphanizomenon ovalisporum

##### Notes

Gkelis et al. 2005a

#### Clastidium rivulare

(Hansgirg) Hansgirg, 1892

Clastidium rivulare

##### Notes

Anagnostidis 1968​

#### Clastidium setigerum

O. Kirchner, 1880

Clastidium setigerum

##### Notes

Anagnostidis 1968​

#### Coelosphaerium kützingianum

Nägeli, 1849

Coelosphaerium minutissimum

##### Notes

Anagnostidis 1968​

#### Coleofasciculus chthonoplastes

(Thuret ex Gomont) M. Siegesmund, J. R. Johansen & T. Friedl in Siegesmund et al. 2008

Microcoleus chtonoplastes

##### Notes

Anagnostidis 1968​

#### Cuspidothrix issatschenkoi

(Usachev) P. Rajaniemi, Komárek, R. Willame, P. Hrouzek, K. Kastovská, L. Hoffmann & K. Sivonen, 2005

Aphanizomenon issatschenkoi

##### Notes

Moustaka-Gouni 1988

#### Cyanobacterium cedrorum

(Sauvageau) Komárek, Kopecky & Cepák, 1999

Synechococcus cedrorum

##### Notes

Anagnostidis 1968​

#### Cyanobacterium minervae

(J. J. Copeland) Komárek, Kopecky & Cepák, 1999

Synechococcus minervae

##### Notes

Anagnostidis 1961​

#### Cyanocatena planctonica

Hindák, 1975

Cyanocatena planctonica

##### Notes

Moustaka-Gouni 1988

#### Cyanodictyon imperfectum

Cronberg & Weibull, 1981

Cyanodictyon imperfectum

##### Notes

Moustaka-Gouni and Nikolaidis 1990

#### Cyanodictyon planctonicum

B. A. Mayer, 1994

Cyanodictyon planctonicum

##### Notes

Tryfon et al. 1996

#### Cyanodictyon reticulatum

(Lemmermann) Geitler, 1925

Cyanodictyon reticulatum

##### Notes

Danielidis et al. 1996

#### Cyanogranis ferruginea

(F. Wawrik) Hindák ex Hindák in Joosen, 2006

Cyanogranis ferruginea

##### Notes

Moustaka-Gouni 1988

#### Cyanosaccus aegaeus

Anagnostidis & Pantazidou, 1985

Cyanosaccus aegaeus

##### Notes

Anagnostidis and Pantazidou 1985

#### Cyanosaccus atticus

Anagnostidis & Pantazidou, 1988

Cyanosaccus atticus

##### Notes

Anagnostidis and Pantazidou 1988b

#### Cyanosarcina burmensis

(Skuja) Kovácik, 1988

Cyanosarcina cf. burmensis

##### Notes

Lamprinou et al. 2012​

#### Cyanosarcina spectabilis

(Geitler) Kovácik, 1988

Cyanosarcina cf. spectabilis

##### Notes

Lamprinou et al. 2012​

#### Cyanosarcina parthenonensis

Anagnostidis in Anagnostidis & Pantazidou, 1991

Cyanosarcina parthenonensis

##### Notes

Lamprinou et al. 2012​

#### Cyanosarcina thalassia

Anagnostidis & Pantazidou, 1991

Cyanosarcina thalassia

##### Notes

Anagnostidis and Pantazidou 1991a

#### Cyanostylon microcystoides

Geitler, 1928

Cyanostylon microcystoides

##### Notes

Anagnostidis 1968​

#### Cyanostylon plancticum

Hindák, 1988

Cyanostylon plancticum

##### Notes

Hindák 1988

#### Cyanothece aeruginosa

(Nägeli) Komárek 1976

Synechococcus aeruginosus

##### Notes

Anagnostidis 1968​

#### Cyanothece halobia

Roussomoustakaki & Anagnostidis 1991

Cyanothece halobia

##### Notes

Anagnostidis and Roussomoustakaki 1991

#### Cyanothece major

(Schröter) Komárek, 1976

Synechococcus maior

##### Notes

Anagnostidis 1968​

#### Cylindrospermopsis raciborskii

(Woloszynska) Seenayya & Subba Raju in Desikachary, 1972

Cylindrospermopsis raciborskii

##### Notes

Moustaka-Gouni 1988

#### Cylindrospermum licheniforme

Kützing ex Bornet & Flahault, 1886

Cylindrospermum licheniforme

##### Notes

Economou-Amilli et al. 1984

#### Cylindrospermum muscicola

Kützing ex Bornet & Flahault, 1886

Cylindrospermum muscicola

##### Notes

Anagnostidis 1968​

#### Dactylococcopsis echini

Rosenvinge in Mortensen & Rosenvinge, 1934

Dactylococcopsis echini

##### Notes

Anagnostidis 1968​

#### Dactylococcopsis rhaphidioides

Hansgirg, 1888

Dactylococcopsis rhaphidioides

##### Notes

Anagnostidis 1968​

#### Dermocarpella prasina

(Reinsch) Komárek & Anagnostidis, 1995

Dermocarpa prasina

##### Notes

Anagnostidis 1968​

#### Dichothrix compacta

Bornet & Flahault, 1886

Dichothrix compacta

##### Notes

Anagnostidis 1968​

#### Dichothrix gypsophila

Bornet & Flahault, 1886

Dichothrix gypsophila

##### Notes

Anagnostidis 1968​

#### Dolichospermum affine

(Lemmermann) Wacklin, L. Hoffmann & Komárek, 2009

Anabaena affinis

##### Notes

Moustaka-Gouni and Nikolaidis 1990

#### Dolichospermum crassum

(Lemmermann) Wacklin, L. Hoffmann & Komárek, 2009

Anabaena crassa

##### Notes

Gkelis et al. 2014

#### Dolichospermum flos-aquae

(Brébisson ex Bornet & Flahault) P. Wacklin, L. Hoffmann & J. Komárek, 2009

Anabaena flos-aquae

##### Notes

Anagnostidis 1968​

#### Dolichospermum lemmermannii

(Richter) P. Wacklin, L. Hoffmann & J. Komárek, 2009

Anabaena lemmermannii var. minor

##### Notes

Tryfon et al. 1997

#### Dolichospermum mendotae

(W. Trelease) Wacklin, L. Hoffmann & Komárek, 2009

Anabaena mendotae

##### Notes

Moustaka-Gouni et al. 2000

#### Dolichospermum mucosum

(Komárková-Legnerová & Eloranta) Wacklin, L. Hoffmann & Komárek, 2009

Anabaena mucosa

##### Notes

Tryfon et al. 1997

#### Dolichospermum scheremetievii

(Elenkin) Wacklin, L. Hoffmann & Komárek, 2009

Anabaena scheremetievii

##### Notes

Anagnostidis 1968​

#### Dolichospermum sigmoideum

(Nygaard) Wacklin, L. Hoffmann & Komárek, 2009

Anabaena circinalis

##### Notes

Moustaka-Gouni 1988

#### Dolichospermum solitarium

(Klebahn) Wacklin, L. Hoffmann & Komárek, 2009

Anabaena solitaria

##### Notes

Hindák 1993

#### Dolichospermum spiroides

(Klebhan) Wacklin, L. Hoffmann & Komárek, 2009

Anabaena spiroides

##### Notes

Anagnostidis 1968​

#### Dolichospermum viguieri

(Denis & Frémy) Wacklin, L. Hoffmann & Komárek, 2009

Anabaena viguieri

##### Notes

Lanaras et al. 1989

#### Entophysalis deusta

(Meneghini) F. E. Drouet & W. A. Daily 1948:

Entophysalis deusta

##### Notes

Anagnostidis 1968​

#### Entophysalis granulosa

Kützing 1843

Entophysalis granulosa

##### Notes

Anagnostidis and Golubic 1966

#### Epigloeosphaera glebulenta

(Zalessky) J. Komárková-Legnerová, 1991

Epigloeosphaera cf. grebulenta

##### Notes

Danielidis et al. 1996

#### Eucapsis terrestris

M. Akiyama, 1965

Eucapsis cf. terrestris

##### Notes

Lamprinou et al. 2012​

#### Eucapsis minor

(Skuja) Elenkin, 1933

Eucapsis minor

##### Notes

Anagnostidis and Economou-Amilli 1978

#### Geitlerinema acutissimum

(Kufferath) Anagnostidis, 1989

Oscillatoria acutissima

##### Notes

Anagnostidis and Golubic 1966

#### Geitlerinema amphibium

(C. Agardh ex Gomont) Anagnostidis, 1989

Oscillatoria amphibia f. circinata

##### Notes

Anagnostidis 1961​

#### Geitlerinema apolloniae

Anagnostidis, 2001

Oscillatoria splendida f. major

##### Notes

Anagnostidis 1961​

#### Geitlerinema lemmermannii

(Woloszynska) Anagnostidis, 1989

Oscillatoria cf. lemmermannii

##### Notes

Hindak and Moustaka 1988

#### Geitlerinema ionicum

(Skuja) Anagnostidis, 1989

Oscillatoria ionica

##### Notes

Anagnostidis et al. 1981

#### Geitlerinema lemmermannii

(Woloszynska) Anagnostidis, 1989

Jaaginema cf. lemmermannii

##### Notes

Moustaka-Gouni 1993

#### Geitlerinema numidicum

(Gomont) Anagnostidis, 1989

Oscillatoria numidica

##### Notes

Anagnostidis 1961​

#### Geitlerinema splendidum

(Greville ex Gomont) Anagnostidis, 1989

Oscillatoria splendida

##### Notes

Anagnostidis and Golubic 1966

#### Geitlerinema sulphureum

(Strzeszewski) Anagnostidis 2001

Oscillatoria geminata f. sulphurea

##### Notes

Anagnostidis 1961​

#### Glaucospira laxissima

(G.S.West) Simic, Komárek & Dordevic, 2014

Spirulina laxissima

##### Notes

Anagnostidis 1961​

#### Gloeobacter violaceus

Rippka, J. B. Waterbury & Cohen-Bazire 1974

Aphanothece caldariorum

##### Notes

Anagnostidis 1961​

#### Gloeocapsa aeruginosa

Kützing, 1843

Gloeocapsa aeruginosa

##### Notes

Lamprinou et al. 2014

#### Gloeocapsa arenaria

(Hassall) Rabenhorst, 1865

Gloeocapsa arenaria

##### Notes

Anagnostidis 1961​

#### Gloeocapsa atrata

Kützing, 1843

Gloeocapsa montana

##### Notes

Economou-Amilli et al. 1984

#### Gloeocapsa biformis

Ercegovic, 1925

Gloeocapsa biformis

##### Notes

Anagnostidis 1968​

#### Gloeocapsa bituminosa

(Bory) Kützing, 1849

Gloeocapsa bituminosa

##### Notes

Lamprinou et al. 2012

#### Gloeocapsa caldariorum

Rabenhorst, 1865

Gloeocapsa caldariorum

##### Notes

Lamprinou et al. 2012​

#### Gloeocapsa compacta

Kützing, 1847

Gloeocapsa compacta

##### Notes

Anagnostidis 1968​

#### Gloeocapsa decorticans

(A. Braun) P.Richter in Wille, 1925

Gloeocapsa decorticans

##### Notes

Anagnostidis et al. 1983

#### Gloeocapsa gelatinosa

Kützing, 1843

Gloeocapsa gelatinosa

##### Notes

Anagnostidis 1961​

#### Gloeocapsa kuetzingiana

Nägeli ex Kützing, 1849

Gloeocapsa

##### Notes

Anagnostidis 1968

#### Gloeocapsa punctata

Nägeli, 1849

Gloeocapsa punctata

##### Notes

Lamprinou et al. 2012​

#### Gloeocapsa rupestris

Kützing, 1847

Gloeocapsa rupestris

##### Notes

Anagnostidis 1961​

#### Gloeocapsa sanguinea

(C. Agardh) Kützing, 1843

Gloeocapsa sanguinea

##### Notes

Anagnostidis 1968​

#### Gloeocapsa thermalis

Kützing, 1843

Gloeocapsa thermalis

##### Notes

Anagnostidis 1968​

#### Gloeocapsa thermophila

(Wood) Claus, 1959

Chroococcus thermophilus

##### Notes

Anagnostidis 1961​

#### Gloeocapsopsis crepidinum

(Thuret) Geitler ex Komárek, 1993

Gloeocapsopsis crepidinum

##### Notes

Lamprinou et al. 2012​

#### Gloeocapsopsis cyanea

(Krieger) Komárek & Anagnostidis, 1995

Gloeocapsopsis cyanea

##### Notes

Lamprinou et al. 2012

#### Gloeocapsopsis pleurocapsoides

(Novácek) Komárek & Anagnostidis ex Komárek, 1993

Gloeocapsopsis pleurocapsoides

##### Notes

Lamprinou et al. 2012​

#### Gloeothece incerta

Skuja, 1964

Gloeothece cf. incerta

##### Notes

Lamprinou et al. 2014

#### Gloeothece tepidariorum

(A. Braun) Lagerheim 1883

Gloeothece cf. tepidariorum

##### Notes

Lamprinou et al. 2014

#### Gloeothece confluens

Nägeli, 1849

Gloeothece confluens

##### Notes

Anagnostidis 1968​

#### Gloeothece fusco-lutea

(Nägeli ex Kützing) Nägeli, 1849

Gloeothece fusco-lutea

##### Notes

Anagnostidis 1968​

#### Gloeothece palea

(Kützing) Nägeli, 1849

Gloeothece palea

##### Notes

Anagnostidis and Golubic 1966

#### Gloeothece rupestris

(Lyngbye) Bornet in Wittrock & Nordstedt, 1880

Gloeothece rupestris

##### Notes

Anagnostidis 1961

#### Gloeothece tepidariorum

(A. Braun) Lagerheim, 1883

Gloeothece rupestris var. tepidariorum

##### Notes

Anagnostidis 1961​

#### Gloeotrichia echinulata

P. G. Richter, 1894

Gloeotrichia echinulata

##### Notes

Anagnostidis 1968​

#### Gloeotrichia pisum

Thuret ex Bornet & Flahault, 1886

Gloeotrichia pisum

##### Notes

Anagnostidis 1968​

#### Gomphosphaeria aponina

Kützing, 1836

Gomphosphaeria aponina

##### Notes

Anagnostidis 1968​

#### Hapalosiphon intricatus

W. & G. S. West, 1894

Hapalosiphon intricatus

##### Notes

Anagnostidis 1968​

#### Hassallia byssoidea

Hassall ex Bornet & Flahault, 1886

Tolypothrix byssoidea

##### Notes

Anagnostidis 1968​

#### Herpyzonema pulverulentum

M. Hernández-Mariné & T. Canals, 1994

Herpyzonema pulverulentum

##### Notes

Lamprinou et al. 2012

#### Heteroleibleinia infixa

(Frémy) Anagnostidis & Komárek, 1988

Lyngbya infixa

##### Notes

Anagnostidis 1968​

#### Heteroleibleinia kuetzingii

(Schmidle) Compère, 1985

Lyngbya kutzingii

##### Notes

Anagnostidis 1968​

#### Homoeothrix caespitosa

Kirchner, 1898

Homoeothrix caespitosa

##### Notes

Anagnostidis 1968

#### Homoeothrix juliana

(Bornet & Flahault ex Gomont) Kirchner,1898

Homoeothrix juliana

##### Notes

Anagnostidis 1968​

#### Hormoscilla feldmannii

Anagnostidis, 2001

Borzia spongeliae

##### Notes

Anagnostidis 1977

#### Hydrococcus cesatii

Rabenhorst, 1860

Hydrococcus cesatii

##### Notes

Anagnostidis 1968​

#### Hydrococcus rivularis

Kützing, 1833

Hydrococcus rivularis

##### Notes

Anagnostidis 1968​

#### Hydrocoleum homoeotrichum

Kützing ex Gomont, 1892

Hydrocoleum homoeotrichum

##### Notes

Anagnostidis 1968​

#### Hydrocoleum muscicola

Hansgirg ex Forti, 1907

Hydrocoleum muscicolum

##### Notes

Anagnostidis 1968

#### Hydrocoleum stankovicii

Čado, 1958

Hydrocoleum stankovicii

##### Notes

Lamprinou et al. 2012​

#### Hyella balani

Lehmann, 1903

Hyella balani

##### Notes

Anagnostidis and Pantazidou 1985

#### Hyella caespitosa

Bornet & Flahault, 1888

Hyella caespitosa

##### Notes

Anagnostidis and Pantazidou 1988a

#### Hyella fontana

Huber & Jadin, 1892

Hyella fontana

##### Notes

Anagnostidis and Pantazidou 1988c

#### Hyella inconstans

Al-Thukair & Golubic 1991

Hyella inconstans

##### Notes

Pantazidou et al. 2006

#### Hyella kalligrammos

Anagnostidis & Pantazidou, 1988

Hyella kalligrammos

##### Notes

Anagnostidis and Pantazidou 1988c

#### Hyella maxima

(Geitler) Anagnostidis & Pantazidou, 1988

Hyella maxima

##### Notes

Anagnostidis and Pantazidou 1988c

#### Hyella reptans

Al-Thukair & Golubic, 1991

Hyella reptans

##### Notes

Pantazidou et al. 2006

#### Ifinoe spelaea

Lamprinou & Pantazidou in Lamprinou et al. 2011

spelaeobios

##### Notes

Lamprinou et al. 2011

#### Isactis plana

Thuret ex Bornet & Flahault, 1886

Calothrix plana

##### Notes

Anagnostidis and Golubic 1966

#### Isocystis halobia

K. Anagnostidis & M. Roussomoustakaki, 1991

Isocystis halobia

##### Notes

Anagnostidis and Roussomoustakaki 1991

#### Isocystis pallida

(Voronichin) Woronichin, 1927

Isocystis pallida

##### Notes

Anagnostidis 1961​

#### Jaaginema angustissimum

(W. & G. S. West) Anagnostidis & Komárek, 1988

Oscillatoria angustissima

##### Notes

Anagnostidis 1961​

#### Jaaginema geminatum

(Schwabe ex Gomont) Anagnostidis & Komárek 1988

Oscillatoria geminata

##### Notes

Anagnostidis 1961​

#### Jaaginema mauchanum

(Claus) Anagnostidis & Komárek, 1988

Oscillatoria cf. mauchiana

##### Notes

Lamprinou et al. 2012​

#### Jaaginema minimum

(Gicklhorn) Anagnostidis & Komárek, 1988

Oscillatoria minima

##### Notes

Anagnostidis 1968

#### Jaaginema profundum

(Schröter & Kirchner) Anagnostidis & Komárek, 1988

Achroonema projundum

##### Notes

Anagnostidis and Economou–Amilli 1980

#### Jaaginema pseudogeminatum

(G. Schmid) Anagnostidis & Komárek, 1988

Oscillatoria pseudogeminata

##### Notes

Anagnostidis and Golubic 1966

#### Jaaginema subtilissimum

(Kützing ex Forti) Anagnostidis & Komárek, 1988

Oscillatoria subtilissima

##### Notes

Anagnostidis 1961​

#### Johanseninema constrictum

(Szafer) Hasler, Dvorák & Poulícková, 2014

Pseudanabaena constricta

##### Notes

Anagnostidis and Economou–Amilli 1980

#### Kamptonema animale

(C. Agardh ex Gomont) Strunecký, Komárek & J. Smarda, 2014

Oscillatoria animalis

##### Notes

Anagnostidis 1961​

#### Kamptonema chlorinum

(Kützing ex Gomont) Strunecký, Komárek & J. Smarda, 2014

Oscillatoria chlorina

##### Notes

Anagnostidis 1968​

#### Kamptonema cortianum

(Meneghini ex Gomont) Strunecký, Komárek & J. Smarda, 2014

Oscillatoria cortiana

##### Notes

Anagnostidis 1961​

#### Kamptonema formosum

(Bory ex Gomont) Strunecký, Komárek & J. Smarda, 2014

Oscillatoria formosa

##### Notes

Anagnostidis 1961​

#### Kamptonema jasorvense

(Vouk) Strunecký, Komárek & J. Smarda, 2014

Oscillatoria jasorvensis

##### Notes

Anagnostidis and Golubic 1966

#### Kamptonema laetevirens

(H.M.Crouan & P.L.Crouan ex Gomont) Strunecký, Komárek & J. Smarda, 2014

Phormidium laetevirens

##### Notes

Anagnostidis and Roussomoustakaki 1988

#### Kamptonema okenii

(C.Agardh ex Gomont) Strunecký, Komárek & J. Smarda, 2014

Oscillatoria okenii

##### Notes

Anagnostidis 1961​

#### Komvophoron crassum

(Vozzennikova) Anagnostidis & Komárek, 1988

Komvophoron cf. crassum

##### Notes

Anagnostidis and Roussomoustakaki 1991

#### Komvophoron pallidum

(Skuja) Anagnostidis & Komárek, 1988

Pseudanabaena pallida

##### Notes

Anagnostidis and Economou–Amilli 1980

#### Komvophoron schmidlei

(Jaag) Anagnostidis & Komárek, 1988

Pseudanabaena schmidlei

##### Notes

Anagnostidis 1968​

#### Kyrtuthrix dalmatica

Ercegović, 1929

Kyrtuthrix dalmatica

##### Notes

Anagnostidis 1968​

#### Leibleinia gracilis

(Rabenhorst ex Gomont) Anagnostidis & Komárek, 1988

Lyngbya gracilis

##### Notes

Anagnostidis and Golubic 1966

#### Leibleinia nordgaardii

Anagnostidis & Komárek, 1988

Lyngbya nordagardhii

##### Notes

Anagnostidis 1968

#### Lemmermanniella pallida

(Lemmermann) Geitler, 1942

Lemmermanniella pallida

##### Notes

Tryfon et al. 1997

#### Leptolyngbya amplivaginata

(van Goor) Anagnostidis & Komárek, 1988

Lyngbya amplivaginata

##### Notes

Anagnostidis 1968​

#### Leptolyngbya angustissima

(W. & G. S. West) Anagnostidis & Komárek, 1988

Phormidium angustissimum

##### Notes

Anagnostidis 1961​

#### Leptolyngbya antarctica

(W. & G. S. West) Anagnostidis & Komárek, 1988

Phormidium antarcticum

##### Notes

Anagnostidis et al. 1982

#### Leptolyngbya boryana

(Gomont) Anagnostidis & Komárek, 1988

Plectonema boryanum

##### Notes

Anagnostidis et al. 1981

#### Leptolyngbya calotrichoides

(Gomont) Anagnostidis & Komárek, 1988

Plectonema calotrichoides

##### Notes

Anagnostidis 1968

#### Leptolyngbya cebennensis

(Gomont) I. Umezaki & M. Watanabe, 1994

Leptolyngbya cebennensis

##### Notes

Lamprinou et al. 2012

#### Leptolyngbya carnea

(Kützing ex Lemmermann) Anagnostidis & Komárek, 1988

Leptolyngbya cf. carnea

##### Notes

Lamprinou et al. 2012

#### Leptolyngbya laminosa

(Gomont ex Gomont) Anagnostidis & Komárek, 1988

Leptolyngbya cf. laminosa

##### Notes

Lamprinou et al. 2014

#### Leptolyngbya compacta

(Kützing ex Hansgirg) Komárek in Anagnostidis, 2001

Leptolyngbya compacta

##### Notes

Lamprinou et al. 2012

#### Leptolyngbya ectocarpi

(Gomont) Anagnostidis & Komárek, 1988

Phormidium ectocarpi

##### Notes

Anagnostidis 1968​

#### Leptolyngbya ercegovicii

(Čado) Anagnostidis & Komárek, 1988

Leptolyngbya ercegovicii

##### Notes

Lamprinou et al. 2012

#### Leptolyngbya foveolara

(Gomont) Anagnostidis & Komárek, 1988

Phormidium foveolarum

##### Notes

Anagnostidis 1961​

#### Leptolyngbya fragilis

(Gomont) Anagnostidis & Komárek, 1988

Phormidium fragile

##### Notes

Anagnostidis 1961​

#### Leptolyngbya gracillima

(Hansgirg) Anagnostidis & Komárek, 1988

Plectonema gracillimum

##### Notes

Anagnostidis et al. 1982

#### Leptolyngbya halophila

(Hansgirg ex Gomont) Anagnostidis & Komárek, 1988

Lyngbya halophila

##### Notes

Anagnostidis 1968​

#### Leptolyngbya lagerheimii

(Gomont ex Gomont) Anagnostidis & Komárek, 1988

Lyngbya lagerheimii

##### Notes

Anagnostidis 1961​

#### Leptolyngbya laminosa

(Gomont ex Gomont) Anagnostidis & Komárek, 1988

Phormidium laminosum

##### Notes

Anagnostidis 1961​

#### Leptolyngbya lurida

(Gomont) Anagnostidis & Komárek, 1988

Phormidium luridum

##### Notes

Anagnostidis 1961​

#### Leptolyngbya nana

(Tilden) Anagnostidis & Komárek, 1988

Leptolyngbya nana

##### Notes

Lamprinou et al. 2012​

#### Leptolyngbya norvegica

(Gomont) Anagnostidis & Komárek, 1988

Plectonema norvegicum

##### Notes

Anagnostidis 1968​

#### Leptolyngbya nostocorum

(Bornet ex Gomont) Anagnostidis & Komárek, 1988

Plectonema nostocorum

##### Notes

Anagnostidis and Golubic 1966

#### Leptolyngbya notata

(Schmidle) Anagnostidis & Komárek, 1988

Plectonema notatum

##### Notes

Anagnostidis 1968​

#### Leptolyngbya ochracea

(Thuret ex Gomont) Anagnostidis & Komárek 1988

Lyngbya ochracea

##### Notes

Anagnostidis 1968​

#### Leptolyngbya perelegans

(Lemmermann) Anagnostidis & Komárek, 1988

Lyngbya perelegans

##### Notes

Anagnostidis and Golubic 1966

#### Leptolyngbya perforans

(Geitler) Anagnostidis & Komárek, 1988

Schizothrix perforans st. typicus

##### Notes

Anagnostidis 1968​

#### Leptolyngbya phormidioides

(Anagnostidis) Anagnostidis & Komárek, 1988

Oscillatoria acuminata f. phormidioides

##### Notes

Anagnostidis 1961​

#### Leptolyngbya purpurascens

(Gomont ex Gomont) Anagnostidis & Komárek, 1988

Leptolyngbya purpurascens

##### Notes

Lamprinou et al. 2012​

#### Leptolyngbya rivulariarum

(Gomont) Anagnostidis & Komárek 1988

Lyngbya rivulariarum

##### Notes

Anagnostidis 1968​

#### Leptolyngbya subtilissima

(Hansgirg) Komárek in Anagnostidis, 2001

Leptolyngbya subtilissima

##### Notes

Lamprinou et al. 2014

#### Leptolyngbya subuliformis

(Gomont) Anagnostidis & Komárek, 1988

Phormidium subuliforme

##### Notes

Anagnostidis 1961​

#### Leptolyngbya tenuis

(Gomont) Anagnostidis & Komárek, 1988

Phormidium tenue f. circinata

##### Notes

Anagnostidis 1961​

#### Leptolyngbya terebrans

(Bornet & Flahault ex Gomont) Anagnostidis & Komárek, 1988

Plectonema terebrans

##### Notes

Anagnostidis and Golubic 1966

#### Leptolyngbya truncata

(Lemmermann) Anagnostidis & Komárek, 1988

Phormidium truncatum

##### Notes

Anagnostidis 1961​

#### Leptolyngbya undosa

(Čado) Anagnostidis & Komárek, 1988

Leptolyngbya undosa

##### Notes

Lamprinou et al. 2012​

#### Leptolyngbya valderiana

(Gomont) Anagnostidis & Komárek 1988

Phormidium valderianum

##### Notes

Anagnostidis 1961

#### Leptolyngbya weedii

(Drouet) Anagnostidis 2001

Phormidium weedii

##### Notes

Anagnostidis and Economou-Amilli 1978

#### Limnococcus limneticus

(Lemmermann) Komárková, Jezberová, O. Komárek & Zapomelová, 2010

Chroococcus limneticus

##### Notes

Anagnostidis 1968​

#### Limnothrix brachynema

(Skuja) Hindák & Trifonova, 1989

Lyngbya brachynema

##### Notes

Anagnostidis 1968​

#### Limnothrix obliqueacuminata

(Skuja) Meffert 1988

Oscillatoria obliqueacuminata

##### Notes

Anagnostidis 1968

#### Limnothrix redekei

(Goor) Meffert, 1988

Limnothrix redekei

##### Notes

Vardaka et al. 2000

#### Lyngbyaf.minor

Elenkin, 1949

Lyngbya aerugineo-coerulea f. minor

##### Notes

Anagnostidis 1961​

#### Lyngbya aestuarii

Liebman ex Gomont, 1892

Lyngbya aestuarii

##### Notes

Anagnostidis 1968

#### Lyngbya agardhii

Gomont, 1892

Lyngbya agardhii

##### Notes

Anagnostidis and Golubic 1966

#### Lyngbya boryana

Kirchner ex Hansgirg, 1892

Phormidium boryanum

##### Notes

Anagnostidis 1961​

#### Lyngbya spiralis

Geitler, 1932

Lyngbya cf. spiralis

##### Notes

Overbeck et al. 1982

#### Lyngbya confervoides

C. Agardh ex Gomont, 1892

Lyngbya confervoides

##### Notes

Anagnostidis and Golubic 1966

#### Lyngbya lutea

Gomont ex Gomont, 1892

Lyngbya lutea

##### Notes

Anagnostidis and Golubic 1966

#### Lyngbya majuscula

Harvey ex Gomont, 1892

Lyngbya majuscula

##### Notes

Anagnostidis 1968​

#### Lyngbya martensiana

Meneghini ex Gomont, 1892

Lyngbya martensiana

##### Notes

Anagnostidis 1961​

#### Lyngbya palikiana

Claus, 1955

Leptolyngbya palikiana

##### Notes

Lamprinou et al. 2012​

#### Lyngbya semiplena

J. Agardh ex Gomont, 1892

Lyngbya semiplena

##### Notes

Anagnostidis and Golubic 1966

#### Lyngbya sordida

Gomont, 1892

Lyngbya sordida

##### Notes

Anagnostidis and Golubic 1966

#### Mastigocladus laminosus

Cohn ex Kirchner, 1898

Mastigocladus laminosus

##### Notes

Anagnostidis 1961​

#### Mastigocoleus testarum

Lagerheim ex Bornet & Flahault, 1887

Mastigocoleus testarum

##### Notes

Anagnostidis 1968​

#### Merismopedia elegans

A. Braun ex Kützing, 1849

Merismopedia elegans

##### Notes

Anagnostidis 1968

#### Merismopedia glauca

(Ehrenberg) Kützing, 1845

Merismopedia glauca

##### Notes

Anagnostidis 1968

#### Merismopedia mediterranea

Nägeli, 1849

Merismopedia glauca f. mediterranea

##### Notes

Anagnostidis and Golubic 1966

#### Merismopedia minima

G. Beck 1897

Merismopedia minima

##### Notes

Anagnostidis 1968​

#### Merismopedia punctata

Meyen, 1839

Merismopedia punctata

##### Notes

Anagnostidis 1968​

#### Merismopedia smithii

De Toni, 1939

Merismopedia maior

##### Notes

Anagnostidis 1968​

#### Merismopedia tenuissima

Lemmermann 1898

Merismopedia tenuissima

##### Notes

Anagnostidis 1968​

#### Merismopedia trolleri

Bachmann, 1920

Merismopedia trolleri

##### Notes

Anagnostidis 1968

#### Merismopedia warmingiana

(Lagerheim) Forti, 1883

Merismopedia warmingiana

##### Notes

Anagnostidis 1968​

#### Microchaete grisea

Thuret ex Bornet & Flahault, 1886

Microchaete grisea

##### Notes

Anagnostidis 1968​

#### Microchaete tenera

Thuret ex Bornet & Flahault, 1886

Microchaete tenera

##### Notes

Anagnostidis 1968​

#### Microcoleus amoenus

(Gomont) Strunecky, Komárek & J. R. Johansen, 2013

Oscillatoria amoena

##### Notes

Anagnostidis 1968​

#### Microcoleus amoenus

(Gomont) Strunecky, Komárek & J. R. Johansen, 2013

Phormidium amoenum

##### Notes

Anagnostidis and Roussomoustakaki 1991

#### Microcoleus autumnalis

(Gomont) Strunecky, Komárek & J. R. Johansen, 2013

Phormidium autumnale

##### Notes

Anagnostidis 1961​

#### Microcoleus ferrugineus

Frémy, 1936

Microcoleus ferrugineus

##### Notes

Anagnostidis 1968​

#### Microcoleus lacustris

Farlow ex Gomont, 1892

Microcoleus lacustris

##### Notes

Anagnostidis 1968​

#### Microcoleus paludosus

Gomont, 1892

Microcoleus paludosus

##### Notes

Anagnostidis 1968​

#### Microcoleus setchellianus

(Gomont) Strunecky, Komárek & J. R. Johansen, 2013

Phormidium setchellianum

##### Notes

Lamprinou et al. 2012

#### Microcoleus steenstrupii

J. B. Petersen, 1923

Microcoleus steenstrupii

##### Notes

Lamprinou et al. 2012​

#### Microcoleus vaginatus

Gomont ex Gomont, 1892

Microcoleus vaginatus

##### Notes

Anagnostidis 1968

#### Microcoleus vulgaris

Strunecky, Komárek & J. R. Johansen 2013

Phormidium vulgare

##### Notes

Lamprinou et al. 2012​

#### Microcystis flos-aquae

(Wittrock) Kirchner, 1898

Microcystis flos-aquae

##### Notes

Anagnostidis 1961​

#### Microcystis aeruginosa

(Kützing) Kützing, 1846

Microcystis aeruginosa

##### Notes

Anagnostidis 1968​

#### Microcystis ichthyoblabe

(G.Kunze) Kützing, 1846

Microcystis ichthyoblabe

##### Notes

Tryfon et al. 1997

#### Microcystis incerta

(Lemmermann) Lemmermann, 1903

Microcystis incerta

##### Notes

Overbeck et al. 1982

#### Microcystis marginata

(Meneghini) Kützing, 1846

Microcystis marginata

##### Notes

Anagnostidis 1968​

#### Microcystis novacekii

(Komárek) Compère, 1974

Microcystis novacekii

##### Notes

Vardaka et al. 2000

#### Microcystis panniformis

Komárek, Komárková-Legnerová, Sant'Anna, M. T. P. Azevedo, & P. A. C. Senna, 2002

Microcystis panniformis

##### Notes

Papadimitriou et al. 2013

#### Microcystis pulverea

(H. C. Wood) Forti, 1907

Microcystis pulverea

##### Notes

Anagnostidis 1968​

#### Microcystis smithii

Komárek & Anagnostidis, 1995

Aphanocapsa pulchra

##### Notes

Anagnostidis 1968​

#### Microcystis viridis

(A. Braun) Lemmermann, 1903

Microcystis viridis

##### Notes

Anagnostidis 1968​

#### Microcystis wesenbergii

(Komárek) Komárek ex Komárek, 1968

Microcystis wesenbergii

##### Notes

Tryfon et al. 1996

#### Myxosarcina concinna

Printz, 1921

Myxosarcina concinna

##### Notes

Anagnostidis et al. 1983

#### Nodularia harveyana

Thuret ex Bornet & Flahault, 1886

Nodularia harveyana

##### Notes

Anagnostidis 1968​

#### Nodularia spumigena

Mertens ex Bornet & Flahault 1888

Nodularia spumigena

##### Notes

Anagnostidis 1968​

#### Nostoc calcicola

Brébisson ex Bornet & Flahault, 1886

Nostoc calcicola

##### Notes

Anagnostidis 1961​

#### Nostoc commune

Vaucher ex Bornet & Flahault, 1888

Nostoc commune

##### Notes

Economou-Amilli et al. 1984

#### Nostoc humifusum

Carmichael ex Bornet & Flahault, 1888

Nostoc humifusum

##### Notes

Anagnostidis 1961​

#### Nostoc letestui

Frémy, 1930

Nostoc letestui

##### Notes

Lamprinou et al. 2012​

#### Nostoc linckia

Bornet ex Bornet & Flahault, 1888

Nostoc linckia

##### Notes

Anagnostidis 1968

#### Nostoc microscopicum

Carmichael ex Bornet & Flahault, 1888

Nostoc microscopicum

##### Notes

Anagnostidis 1968​

#### Nostoc minutissimum

Kützing ex Bornet & Flahault, 1888

Nostoc minutissimum

##### Notes

Anagnostidis 1968​

#### Nostoc paludosum

Kützing ex Bornet & Flahault, 1888

Nostoc paludosum

##### Notes

Anagnostidis 1961​

#### Nostoc punctiforme

Hariot 1891

Nostoc punctiforme

##### Notes

Anagnostidis et al. 1981

#### Nostoc sphaericum

Vaucher ex Bornet & Flahault, 1888

Nostoc sphaericum

##### Notes

Anagnostidis 1968​

#### Oculatella subterranea

Zammit, Billi & Albertano, 2012

Oculatella subterranea

##### Notes

Christodoulou et al. 2015

#### Oscillatoria anguina

Bory ex Gomont 1892

Oscillatoria anguina

##### Notes

Anagnostidis 1961​

#### Oscillatoria bonnemaisonii

P. Crouan & H. Crouan ex Gomont, 1892

Oscillatoria bonnemaisonii

##### Notes

Anagnostidis 1968​

#### Oscillatoria corallinae

Gomont ex Gomont, 1892

Oscillatoria corallinae

##### Notes

Anagnostidis and Golubic 1966

#### Oscillatoria crassa

(C. B. Rao) Anagnostidis, 2001

Oscillatoria ornata var. crassa

##### Notes

Anagnostidis 1961​

#### Oscillatoria curviceps

C. Agardh ex Gomont, 1892

Oscillatoria curviceps

##### Notes

Anagnostidis 1968

#### Oscillatoria laetevirens

Hofman-Bang ex Forti 1892

Oscillatoria laetevirens

##### Notes

Anagnostidis 1961​

#### Oscillatoria limosa

C. Agardh ex Gomont 1892

Oscillatoria limosa

##### Notes

Anagnostidis and Golubic 1966

#### Oscillatoria margaritifera

Kützing ex Gomont, 1892

Oscillatoria margaritifera

##### Notes

Anagnostidis 1968​

#### Oscillatoria okeniivar.gracilis

(Kützing) Kützing ex Forti, 1904

Oscillatoria okenii f. gracilis

##### Notes

Anagnostidis 1961​

#### Oscillatoria ornata

Kützing ex Gomont, 1892

Oscillatoria ornata

##### Notes

Anagnostidis 1961​

#### Oscillatoria princeps

Vaucher ex Gomont, 1892

Oscillatoria princeps

##### Notes

Anagnostidis 1961​

#### Oscillatoria pseudoangusta

Claus, 1955

Oscillatoria pseudoangusta

##### Notes

Anagnostidis 1961​

#### Oscillatoria putrida

Schmidle, 1901

Oscillatoria putrida

##### Notes

Anagnostidis 1968

#### Oscillatoria rhamphoidea

Anagnostidis, 2001

Oscillatoria salina f. major

##### Notes

Anagnostidis et al. 1981

#### Oscillatoria rupicola

(Hansgirg) Hansgirg ex Forti, 1907

Oscillatoria rupicola

##### Notes

Lamprinou et al. 2012​

#### Oscillatoria sancta

Kützing ex Gomont 1892

Oscillatoria sancta

##### Notes

Anagnostidis 1961​

#### Oscillatoria simplicissima

Gomont, 1892

Oscillatoria simplicissima

##### Notes

Anagnostidis 1961​

#### Oscillatoria subbrevis

Schmidle, 1901

Oscillatoria subbrevis

##### Notes

Anagnostidis et al. 1981

#### Oscillatoria tenuis

C. Agardh ex Gomont, 1892

Oscillatoria tenuis

##### Notes

Anagnostidis 1961​

#### Oscillatoriavar.phormidioides

Hansgirg ex Drouet, 1957

Oscillatoria okenii f. phormidioides

##### Notes

Anagnostidis 1961​

#### Oscillatoria trichoides

Szafer, 1910

Oscillatoria trichoides

##### Notes

Anagnostidis and Golubic 1966

#### Oxynema acuminatum

(Gomont) Chatchawan, Komárek, Strunecky, Smarda & Peerapornpisal, 2012

Oscillatoria acuminata

##### Notes

Anagnostidis 1961​

#### Pannus spumosus

B.Hickel, 1991

Pannus spumosus

##### Notes

Hindak and Moustaka 1988

#### Petalonema densum

(Bornet ex Bornet & Flahault) Migula, 1907

Petalonema densum

##### Notes

Anagnostidis 1968

#### Phormidesmis molle

(Gomont) Turicchia, Ventura, Komárková & Komárek, 2009

Phormidium molle

##### Notes

Anagnostidis 1961​

#### Phormidiochaete nordstedtii

(Bornet et Flahault) Komárek in Anagnostidis, 2001

Phormidiochaete nordstedtii

##### Notes

Lamprinou et al. 2012​

#### Phormidium acuminatumvar.longe-attenuatum

(Geitler & Ruttner) I. Umezaki & M. Watanabe, 1994

Oscillatoria acuminata f. longe-attenuata

##### Notes

Anagnostidis 1961

#### Phormidium aerugineo-caeruleum

(Gomont) Anagnostidis & Komárek, 1988

Lyngbya aerugineo-coerulea

##### Notes

Anagnostidis 1961​

#### Phormidium ambiguum

Gomont, 1892

Phormidium ambiguum

##### Notes

Anagnostidis 1961​

#### Phormidium articulatum

(N. L. Gardner) Anagnostidis & Komárek, 1988

Phormidium articulatum

##### Notes

Lamprinou et al. 2012​

#### Phormidium boryanum

Kützing, 1843

Oscillatoria boryana

##### Notes

Anagnostidis and Golubic 1966

#### Phormidium breve

(Kützing ex Gomont) Anagnostidis & Komárek, 1988

Oscillatoria brevis

##### Notes

Anagnostidis 1961​

#### Phormidium griseoviolaceum

(Skuja) Anagnostidis, 2001

Phormidium cf. griseoviolaceum

##### Notes

Lamprinou et al. 2012​

#### Phormidium chalybeum

(Mertens ex Gomont) Anagnostidis & Komárek, 1988

Oscillatoria chalybea

##### Notes

Anagnostidis 1961​

#### Phormidium corium

Gomont ex Gomont, 1892

Phormidium corium

##### Notes

Anagnostidis 1961​

#### Phormidium diguetii

(Gomont) Anagnostidis & Komárek, 1988

Lyngbya diguetii

##### Notes

Anagnostidis 1961​

#### Phormidium edessae

Skuja, 1937

Phormidium edessae

##### Notes

Anagnostidis 1968​

#### Phormidium endophyticum

(W. & G. S. West) Skuja, 1956

Phormidium endophyticum

##### Notes

Anagnostidis 1968​

#### Phormidium favosum

Gomont, 1892

Phormidium favosum

##### Notes

Anagnostidis 1968

#### Phormidium feldmanni

Frémy, 1937

Phormidium feldmanni

##### Notes

Anagnostidis 1968​

#### Phormidium grunowianum

(Gomont) Anagnostidis & Komárek, 1988

Oscillatoria grunowiana

##### Notes

Anagnostidis 1961​

#### Phormidium henningsii

(Lemmermann) Anagnostidis, 2001

Leptolyngbya henningsii

##### Notes

1. Lamprinou et al. 2012

#### Phormidium incrustatum

Gomont ex Gomont, 1892

Phormidium incrustans

##### Notes

Anagnostidis 1968​

#### Phormidium interruptum

Kützing ex Gomont, 1892

Phormidium interruptum

##### Notes

Lamprinou et al. 2012

#### Phormidium inundatum

Kützing ex Gomont, 1892

Phormidium inundatum

##### Notes

Anagnostidis 1961​

#### Phormidium janthiphorum

Elenkin, 1949

Oscillatoria janthiphora

##### Notes

Anagnostidis and Golubic 1966

#### Phormidium jenkelianum

Kützing ex Gomont, 1892

Phormidium jenkelianum

##### Notes

Anagnostidis 1961​

#### Phormidium kuetzingianum

(Kirchner ex Gomont) Anagnostidis & Komárek, 1988

Phormidium kuetzingianum

##### Notes

Lamprinou et al. 2012

#### Phormidium lacustre

(Čado) Anagnostidis, 2001

Phormidium lacustre

##### Notes

Lamprinou et al. 2012​

#### Phormidium litorale

Golubic, 1960

Phormidium litorale

##### Notes

Anagnostidis and Golubic 1966

#### Phormidium macedonicum

(Čado), 1959

Phormidium macedonicum

##### Notes

Lamprinou et al. 2012​

#### Phormidium melanochroun

Lamprinou & Pantazidou, 2013

Phormidium melanochroun

##### Notes

Lamprinou et al. 2013b

#### Phormidium minutum

Lindstedt, 1943

Phormidium minutum

##### Notes

Anagnostidis 1968​

#### Phormidium molischii

(Vouk) Anagnostidis & Komárek, 1988

Lyngbya molischi

##### Notes

Anagnostidis 1968

#### Phormidium mollevar.tenuior

W. & G. S. West ex Geitler, 1925

Phormidium molle f. tenuior

##### Notes

Anagnostidis 1961

#### Phormidium nigroviride

(Thwaites ex Gomont) Anagnostidis & Komárek, 1988

Oscillatoria nigroviridis

##### Notes

Anagnostidis and Golubic 1966

#### Phormidium nigrum

(Vaucher ex Gomont) Anagnostidis & Komárek, 1988

Oscillatoria nigra

##### Notes

Anagnostidis 1968​

#### Phormidium pachydermaticum

Frémy, 1930

Phormidium pachydermaticum

##### Notes

Anagnostidis 1961

#### Phormidium papyraceum

Gomont ex Gomont, 1892

Phormidium papyraceum

##### Notes

Anagnostidis 1961​

#### Phormidium priestleyi

F. E. Fritsch, 1917

Phormidium priestleyi

##### Notes

Lamprinou et al. 2012​

#### Phormidium retzii

Kützing ex Gomont, 1892

Phormidium retzii

##### Notes

Anagnostidis 1961​

#### Phormidium subfuscum

Kützing ex Gomont. 1892

Phormidium subfuscum

##### Notes

Anagnostidis et al. 1981

#### Phormidium submembranaceum

Gomont, 1892

Phormidium submembranaceum

##### Notes

Anagnostidis 1968​

#### Phormidium tanganyikae

(G. S. West) Anagnostidis & Komárek, 1988

Oscillatoria tanganyikae

##### Notes

Anagnostidis et al. 1981

#### Phormidium terebriforme

(C. Agardh ex Gomont) Anagnostidis & Komárek, 1988

Oscillatoria terebriformis

##### Notes

Anagnostidis 1961​

#### Phormidium tergestinum

Kützing ex Anagnostidis & Komárek, 1988

Phormidium tergestinum

##### Notes

Lamprinou et al. 2012​

#### Phormidium thwaitesii

I. Umezaki & M. Watanabe, 1994

Oscillatoria subuliformis

##### Notes

Anagnostidis 1968​

#### Phormidium uncinatum

Gomont ex Gomont, 1892

Phormidium uncinatum

##### Notes

Anagnostidis 1968​

#### Phormidium willei

(N. L. Gardner) Anagnostidis & Komárek, 1988

Oscillatoria willei

##### Notes

Anagnostidis and Golubic 1966

#### Planktolyngbya bipunctata

(Lemmermann) Anagnostidis & Komárek, 1988

Lyngbya bipunctata

##### Notes

Anagnostidis and Golubic 1966

#### Planktolyngbya circumcreta

(G. S. West) Anagnostidis & Komárek, 1988

Lyngbya circumcreta

##### Notes

Moustaka-Gouni 1988

#### Planktolyngbya contorta

(Lemmermann) Anagnostidis & Komárek, 1988

Lyngbya contorta

##### Notes

Anagnostidis 1968​

#### Planktolyngbya limnetica

(Lemmermann) Komárková- Legnerová & Cronberg, 1992

Lyngbya limnetica

##### Notes

Anagnostidis 1961​

#### Planktothrix agardhii

(Gomont) Anagnostidis & Komárek, 1988

Planktothrix agardhii

##### Notes

Papadimitriou et al. 2013

#### Planktothrix isothrix

(Skuja) Komárek & Komárková, 2004

Oscillatoria cf. agardhii var. isothrix

##### Notes

Overbeck et al. 1982

#### Planktothrix mougeotii

Anagnostidis & Komárek, 1988

Planktothrix mougeotii

##### Notes

Anagnostidis and Pantazidou 1988c

#### Planktothrix rubescens

(De Candolle ex Gomont) Anagnostidis & Komárek, 1988

Planktothrix rubescens

##### Notes

Vareli et al. 2009

#### Plectonema araucanum

Schwabe, 1960

Plectonema araucanum

##### Notes

Lamprinou et al. 2012

#### Plectonema tomasinianum

Bornet ex Gomont, 1893

Plectonema tomassinianum

##### Notes

Anagnostidis and Economou-Amilli 1978

#### Pleurocapsa fuliginosa

Hauck, 1885

Pleurocapsa fuliginosa

##### Notes

Lamprinou et al. 2012​

#### Pleurocapsa minor

Hansgirg, 1891

Scopulonema minus

##### Notes

Anagnostidis 1968​

#### Porphyrosiphon versicolor

(Gomont) Anagnostidis & Komárek, 1988

Lyngbya versicolor

##### Notes

Anagnostidis 1968​

#### Pseudanabaena amphigranulata

(Van Goor) Anagnostidis, 2001

Pseudanabaena galeata f. tenuis

##### Notes

Anagnostidis 1961​

#### Pseudanabaena arcuata

(Skuja) Anagnostidis & Komárek, 1988

Phormidium arcuatum

##### Notes

Anagnostidis 1968​

#### Pseudanabaena biceps

Böcher, 1946

Pseudanabaena biceps

##### Notes

Anagnostidis 1968

#### Pseudanabaena catenata

Lauterborn, 1915

Pseudanabaena catenata

##### Notes

Anagnostidis 1961​

#### Pseudanabaena galeata

Böcher, 1949

Pseudanabaena galeata

##### Notes

Anagnostidis 1961​

#### Pseudanabaena limnetica

(Lemmermann) Komárek, 1974

Oscillatoria limnetica

##### Notes

Anagnostidis 1968​

#### Pseudanabaena lonchoides

Anagnostidis, 1961

Pseudanabaena lonchoides

##### Notes

Anagnostidis 1961

#### Pseudanabaena mucicola

(Naumann & Huber-Pestalozzi) Schwabe, 1964

Phormidium mucicola

##### Notes

Anagnostidis 1968​

#### Pseudanabaena raphidioides

(Geitler) Anagnostidis & Komárek, 1988

Pseudanabaena raphidioides

##### Notes

Montesanto et al. 1999

#### Pseudocapsa dubia

Ercegovic, 1925

Pseudocapsa dubia

##### Notes

Anagnostidis and Pantazidou 1991a

#### Pseudophormidium battersii

(Gomont) Anagnostidis, 2001

Plectonema battersii

##### Notes

Anagnostidis 1968​

#### Pseudophormidium golenkinianum

(Gomont) Anagnostidis, 2001

Plectonema golenkinianum

##### Notes

Anagnostidis and Golubic 1966

#### Pseudophormidium hollerbachianum

(Elenkin) Anagnostidis, 2001

Plectonema boryanum f. hollerbachianum

##### Notes

Anagnostidis et al. 1983

#### Pseudophormidium radiosum

(Gomont) Anagnostidis & Komárek, 1988

Plectonema radiosum

##### Notes

Anagnostidis 1968​

#### Pseudophormidium spelaeoides

(Čado) Anagnostidis, 2001

Pseudophormidium spelaeoides

##### Notes

Lamprinou et al. 2012​

#### Pseudophormidium tenue

(Thuret ex Gomont) Anagnostidis & Komárek, 1988

Plectonema tenue

##### Notes

Anagnostidis and Economou-Amilli 1978

#### Pseudoscytonema endolithicum

(Ercegovic) Anagnostidis in Komárek & Anagnostidis, 2005

Plectonema endolithicum

##### Notes

Anagnostidis 1968

#### Radiocystis geminata

Skuja, 1948

Radiocystis geminata

##### Notes

Anagnostidis 1968

#### Raphidiopsis mediterranea

Skuja, 1937

Raphidiopsis mediterranea

##### Notes

Anagnostidis 1968​

#### Rhabdoderma curtum

(Setchell) Komárek & Anagnostidis, 1995

Synechococcus curtus

##### Notes

Anagnostidis and Golubic 1966​

#### Rhabdoderma lineare

Schmidle & Lauterborn in Schmidle, 1900

Synechococcus linearis

##### Notes

Moustaka 1988

#### Rhabdogloea elenkinii

(Roll) Komárek & Anagnostidis, 1995

Dactylococcopsis cf. elenkinii

##### Notes

Hindak and Moustaka 1988

#### Rivularia atra

Roth ex Bornet & Flahault, 1886

Rivularia atra

##### Notes

Anagnostidis 1968

#### Rivularia biasolettiana

Meneghini ex Bornet & Flahault, 1886

Rivularia biasoletiana

##### Notes

Anagnostidis 1968

#### Rivularia bullata

Berkeley ex Bornet & Flahault, 1886

Rivularia bullata

##### Notes

Anagnostidis 1968​

#### Rivularia calcarata

(Woronichin) Poljankij in Hollerbach et al. 1953

Dichothrix compacta var. calcarata

##### Notes

Anagnostidis 1968​

#### Rivularia haematites

C. Agardh ex Bornet & Flahault, 1886

Rivularia haematites

##### Notes

Anagnostidis 1968​

#### Rivularia nitida

C. Agardh ex Bornet & Flahault, 1886

Rivularia nitida

##### Notes

Anagnostidis 1968​

#### Rivularia polyotis

Roth ex Bornet & Flahault, 1886

Rivularia polyotis

##### Notes

Anagnostidis and Golubic 1966​

#### Romeria gracilis

(Koczwara) Koczwara in Geitler, 1932

Romeria gracilis

##### Notes

Tryfon et al. 1996

#### Romeria simplex

(Hindák) Hindák, 1988

Romeria simplex

##### Notes

Katsiapi et al. 2012

#### Schizothrix affinis

Lemmermann, 1905

Schizothrix affinis

##### Notes

Anagnostidis 1968​

#### Schizothrix arenaria

Gomont, 1892

Schizothrix arenaria

##### Notes

Anagnostidis 1968​

#### Schizothrix bosniaca

(Forti) Geitler, 1932

Schizothrix bosniaca

##### Notes

Anagnostidis et al. 1983

#### Schizothrix calcicola

Gomont, 1892

Schizothrix calcicola

##### Notes

Anagnostidis 1968​

#### Schizothrix coriacea

Gomont, 1892

Schizothrix coriacea

##### Notes

Anagnostidis 1968​

#### Schizothrix delicatissima

W. & G. S. West, 1897

Schizothrix delicatissima

##### Notes

Anagnostidis 1968​

#### Schizothrix fasciculata

Gomont ex Gomont, 1892

Schizothrix fasciculata

##### Notes

Anagnostidis 1968​

#### Schizothrix heufleri

Grunow ex Gomont, 1892

Schizothrix heufleri

##### Notes

Anagnostidis 1968​

#### Schizothrix lacustris

A. Braun ex Gomont, 1892

Schizothrix lacustris

##### Notes

Anagnostidis 1968

#### Schizothrix lardacea

Gomont, 1892

Schizothrix lardacea

##### Notes

Anagnostidis 1968​

#### Schizothrix lateritia

Gomont, 1892

Schizothrix lateritia

##### Notes

Anagnostidis 1968​

#### Schizothrix lenormandiana

Gomont, 1892

Schizothrix lenormandiana

##### Notes

Katsiapi et al. 2012

#### Schizothrix simplicior

Skuja, 1964

Schizothrix simplicior

##### Notes

Anagnostidis 1968​

#### Schizothrix tenuis

Woronichin, 1923

Schizothrix tenuis

##### Notes

Anagnostidis 1968​

#### Scytonema crispum

Bornet ex De Toni, 1907

Scytonema crispum st. chiastus

##### Notes

Anagnostidis 1968​

#### Scytonema hofmaniivar.calcicola

Agardh ex Bornet & Flahault, 1887

Scytonema hofmanii var. calcicola

##### Notes

Lamprinou et al. 2012​

#### Scytonema julianum

Meneghini ex B. A. Whitton, 2011

Scytonema julianum

##### Notes

Anagnostidis et al. 1982

#### Scytonema myochrous

C. Agardh ex Bornet & Flahault, 1886

Scytonema myochrous

##### Notes

Anagnostidis 1968​

#### Scytonema polycystum

Bornet & Flahault, 1886

Scytonema polycystum

##### Notes

Anagnostidis 1968​

#### Scytonema wolleanum

Forti, 1907

Scytonema mirabile

##### Notes

Anagnostidis 1968​

#### Scytonematopsis crustacea

(Thuret ex Bornet & Flahault) Koválik & Komárek, 1988.

Calothrix crustacea

##### Notes

Anagnostidis and Golubic 1966​

#### Siphonema polonicum

(Raciborski) Geitler, 1925

Siphononema polonicum

##### Notes

Anagnostidis 1968​

#### Snowella atomus

Komárek & Hindák, 1988

Snowella atomus

##### Notes

Danielidis et al. 1996

#### Snowella lacustris

(Chodat) Komárek & Hindák, 1988

Gomphosphaeria lacustris

##### Notes

Anagnostidis 1968

#### Snowella litoralis

(Häyrén) Komárek & Hindák, 1988

Snowella litoralis

##### Notes

Hindak and Moustaka 1988

#### Snowella septentrionalis

Komárek & Hindák, 1988

Snowella septentrionalis

##### Notes

Montesanto et al. 1999

#### Solentia paulocellularis

(Ercegovic) LeCampion-Alsumard & Golubic ex Belyakova, 1988

Hormathonema paulocellulare

##### Notes

Anagnostidis and Pantazidou 1988a

#### Sphaerocavum microcystiforme

(Hindák) Azevedo & Sant' Anna, 2003

Pannus microcystiformis

##### Notes

Hindák 1993

#### Sphaerospermopsis aphanizomenoides

(Forti) Zapomelová, Jezberová, Hrouzek, Hisem, Reháková & Komárková, 2010

Anabaena aphanizomenoides

##### Notes

Moustaka-Gouni 1988

#### Spirulina caldaria

Tilden, 1898

Spirulina caldaria

##### Notes

Anagnostidis 1961​

#### Spirulina corakiana

Playfair, 1914

Spirulina corakiana

##### Notes

Anagnostidis 1961​

#### Spirulina labyrinthiformis

Gomont, 1892

Spirulina labyrinthiformis

##### Notes

Anagnostidis and Golubic 1966​

#### Spirulina major

Kützing ex Gomont, 1892

Spirulina major

##### Notes

Anagnostidis 1961

#### Spirulina meneghiniana

Zanardini ex Gomont, 1892

Spirulina meneghiniana

##### Notes

Anagnostidis and Golubic 1966​

#### Spirulina princeps

W. & G. S. West, 1902

Spirulina princeps

##### Notes

Anagnostidis 1961

#### Spirulina robusta

H. Welsh, 1965

Spirulina subsalsa var. crassior

##### Notes

Anagnostidis 1961​

#### Spirulina subsalsa

Oersted ex Gomont, 1892

Spirulina subsalsa

##### Notes

Anagnostidis 1961​

#### Spirulina subtilissima

Kützing ex Gomont, 1892

Spirulina subtilissima

##### Notes

Anagnostidis 1961​

#### Spirulina tenerrima

Kützing ex Gomont, 1892

Spirulina tenerrima

##### Notes

Anagnostidis 1961​

#### Spirulina versicolor

Cohn ex Gomont, 1892

Spirulina subsalsa st. versicolor

##### Notes

Anagnostidis 1968​

#### Stanieria sublitoralis

(Lindstedt) Anagnostidis & Pantazidou, 1991

Staniera sublitoralis

##### Notes

Anagnostidis and Pantazidou 1991a

#### Stanieria sphaerica

(Setchel & Gardner) Anagnostidis & Pantazidou,1991

Dermocarpa sphaerica

##### Notes

Anagnostidis and Golubic 1966​

#### Stichosiphon pseudopolymorphus

(F. E. Fritsch) Komárek, 1989

Chamaesiphon pseudopolymorphus

##### Notes

Anagnostidis 1968​

#### Stigonema hormoides

Bornet & Flahault, 1886

Stigonema hormoides

##### Notes

Anagnostidis and Economou-Amilli 1978

#### Stigonema mamillosum

C. Agardh ex Bornet & Flahault, 1887

Stigonema mamillosum

##### Notes

Anagnostidis 1968​

#### Stigonema minutum

Hassall ex Bornet & Flahault, 1887

Stigonema minutum

##### Notes

Anagnostidis 1968​

#### Symploca dubia

Gomont, 1892

Symploca dubia

##### Notes

Anagnostidis 1968

#### Symploca elegans

Kützing ex Gomont, 1892

Symploca elegans

##### Notes

Anagnostidis 1968​

#### Symploca hydnoides

Kützing ex Gomont, 1892

Symploca hydnoides

##### Notes

Anagnostidis 1968

#### Symploca lacrimans

Claus, 1962

Symploca lacrimans

##### Notes

Lamprinou et al. 2012​

#### Symploca muralis

Kützing ex Gomont, 1892

Symploca muralis

##### Notes

Lamprinou et al. 2012

#### Symploca muscorum

Gomont ex Gomont, 1892

Symploca muscorum

##### Notes

Anagnostidis 1968

#### Symploca radians

(Kützing) Rabenhorst ex Gomont, 1892

Symploca radians

##### Notes

Lamprinou et al. 2012

#### Symploca thermalis

Gomont, 1892

Symploca thermalis

##### Notes

Anagnostidis 1961

#### Symplocastrum penicillatum

(Gomont) Anagnostidis, 2001

Schizothrix penicillata

##### Notes

Anagnostidis 1968​

#### Synechococcus salinarum

Komárek, 1956

Synechoccus salinarum

##### Notes

Anagnostidis and Roussomoustakaki 1991

#### Synechococcus bigranulatus

Skuja, 1933

Synechococcus elongatus f. thermalis

##### Notes

Anagnostidis 1961

#### Synechococcus elongatus

Nägeli, 1849

Synechococcus elongatus

##### Notes

Anagnostidis and Golubic 1966​

#### Synechocystis aquatilis

Sauvageau, 1892

Synechocystis aquatilis

##### Notes

Anagnostidis 1961​

#### Synechocystis crassa

Woronichin, 1929

Synechocystis crassa

##### Notes

Anagnostidis 1968​

#### Synechocystis diplococca

(Pringsheim) Bourrelly, 1970

Synechocystis diplococcus

##### Notes

Anagnostidis and Economou-Amilli 1978​

#### Synechocystis endobiotica

(Elenkin & Hollerbach) Elenkin, 1938

Aphanocapsa endophytica

##### Notes

Anagnostidis 1968​

#### Synechocystis minuscula

Woronichin, 1926

Synechocystis minuscula

##### Notes

Anagnostidis 1961​

#### Synechocystis pevalekii

Ercegovic 1925

Synechocystis pevalekii

##### Notes

Anagnostidis 1968​

#### Synechocystis salina

Wislouch, 1924

Synechocystis salina

##### Notes

Anagnostidis 1961​

#### Synechocystis sallensis

Skuja, 1930

Synechocystis sallensis

##### Notes

Metaxatos et al. 2003

#### Synechocystis thermalis

J. J. Copeland, 1936

Synechocystis thermalis

##### Notes

Anagnostidis 1961​

#### Tapinothrix crustacea

(Woronichin) Bohunická & Johansen in Bohunická, Johansen & Fuciková, 2011

Homoeothrix crustacea

##### Notes

Anagnostidis 1968​

#### Tapinothrix varians

(Geitler) Bohunická & J. R. Johansen in Bohunická, Johansen & Fuciková, 2011

Homoeothrix varians

##### Notes

Anagnostidis 1968

#### Tolypothrix cavernicola

Weber-van Bosse, 1913

Tolypothrix cavernicola

##### Notes

Lamprinou et al. 2012​

#### Tolypothrix distorta

Kützing ex Bornet & Flahault, 1886

Tolypothrix distorta

##### Notes

Anagnostidis 1968

#### Tolypothrix elenkinii

Hollerbach, 1923

Tolypothrix elenkinii

##### Notes

Anagnostidis 1968​

#### Tolypothrix fragilissima

Ercegovic, 1925

Tolypothrix fragilissima

##### Notes

Lamprinou et al. 2012​

#### Tolypothrix penicillata

Thuret ex Bornet & Flahault, 1886

Tolypothrix penicillata

##### Notes

Anagnostidis 1968​

#### Tolypothrix rivularis

Hansgirg, 1892

Tolypothrix rivularis

##### Notes

Lamprinou et al. 2012​

#### Toxopsis calypsus

Lamprinou, Skaraki, Kotoulas, Economou-Amili & Pantazidou, 2012

Toxopsis calypsus

##### Notes

Lamprinou et al. 2012​

#### Trichocoleus delicatulus

(W. & G. S. West) Anagnostidis, 2001

Microcoleus delicatulus

##### Notes

Anagnostidis 1968​

#### Trichocoleus minimus

(Frémy) Anagnostidis, 2001

Microcoleus minimus

##### Notes

Anagnostidis et al. 1982

#### Trichocoleus sociatus

(W. & G. S. West) Anagnostidis, 2001

Microcoleus sociatus

##### Notes

Anagnostidis 1961​

#### Trichocoleus tenerrimus

(Gomont) Anagnostidis, 2001

Microcoleus tenerrimus

##### Notes

Anagnostidis and Economou-Amilli 1978​

#### Trichocoleus voukii

(Frémy) Anagnostidis, 2001

Microcoleus voukii

##### Notes

Anagnostidis 1968​

#### Trichodesmium iwanoffianum

Nygaard, 1926

Oscillatoria cf. iwanoffiana

##### Notes

Moustaka 1988

#### Trichodesmium erythraeum

Ehrenberg ex Gomont, 1892

Trichodesmium erythraeum

##### Notes

Metaxatos et al. 2003

#### Trichormus azollae

(Strasburger) Komárek & Anagnostidis, 1989

Anabaena azollae

##### Notes

Anagnostidis et al. 1988b

#### Trichormus thermalis

(V.Vouk) Komárek & Anagnostidis, 1989

Anabaena thermalis

##### Notes

Anagnostidis 1961​

#### Trichormus variabilis

(Kützing ex Bornet & Flahault) Komárek & Anagnostidis, 1989

Anabaena variabilis

##### Notes

Anagnostidis 1968

#### Woronichinia compacta

(Lemmermann) Komárek & Hindák, 1988

Woronichinia compacta

##### Notes

Tryfon et al. 1996

#### Woronichinia naegeliana

(Unger) Elenkin, 1933

Coelosphaerium

##### Notes

Anagnostidis 1968​

#### Xenococcus pyriformis

Setchell & N. L. Gardner in N. L. Gardner, 1918

Xenococcus cf. pyriformis
Xenococcus pyriformis Notes: Radea et al. 2010

#### Xenococcus schousboei

Thuret in Bornet & Thuret, 1880

Xenococcus schousboei

##### Notes

Anagnostidis and Golubic 1966​

#### Xenotholos kerneri

(Hansgirg) M. Gold-Morgan, G. Montejano & J. Komárek, 1994

Xenococcus kerneri

##### Notes

Anagnostidis and Golubic 1966

#### Yonedaella lithophila

(Ercegovic) Umezaki, 1962

Sphaeronema lithophilum

##### Notes

Anagnostidis 1968

## Analysis

**Figure 1.**
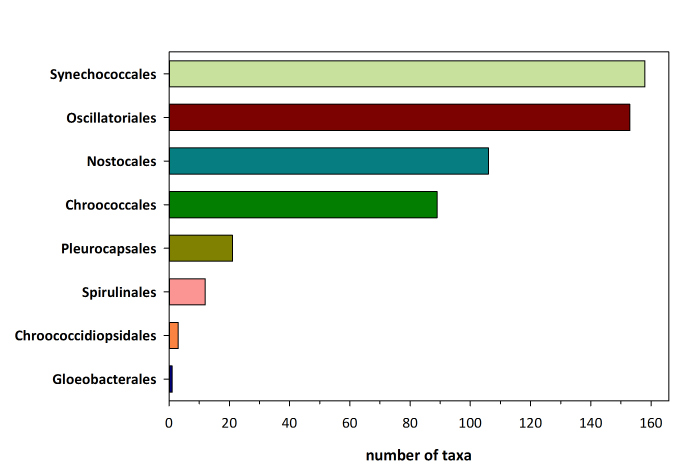
Classification of cyanobacteria of Greece in orders.

A total of 543 species and 85 infraspecific taxa have been found in Greece up to date. The species are classified in 130 genera, 41 families, and 8 orders. The orders Synechococcales and Oscillatoriales have the highest number of species (158 and 153 species, respectively), whereas these two orders along with Nostocales and Chroococcales cover 93% of the known Greek cyanobacteria species (Fig. 1). The families Oscillatoriaceae, Leptolyngbyaceae, Microcoleaceae, and Chroococcaceae have the highest number of species (>30 each) (Fig. 2a); the genera *Phormidium*, *Leptolyngbya*, *Oscillatoria*, *Chroococcus*, *Aphanocapsa*, and *Gloeocapsa* have the highest number of species (>15 each) (Fig. 2b). Fourteen species were found to include 85 infraspecific taxa (Table 1); those infraspecific taxa were not included in the checklist. Furthermore, 21 species recorded in Greece are not valid or of uncertain taxonomic status (Table 2) and were not included in the checklist. 

## Discussion

A thorough review of the relevant literature revealed that 543 species of cyanobacteria have been reported up to date from Greece. The species recorded from this area are classified in 130 genera and 41 families, all of the 8 orders of the class Cyanophyceae (Komárek et al. 2014) being represented. In this checklist we have not included 85 infraspecific taxa mostly from the genera *Entophysalis*, *Gloeocapsa*, *Mastigocladus*, *Scytonema*, and *Siphononema* recorded in several habitats such as gulfs, waterfalls, caves, and lakes by Anagnostidis (Anagnostidis 1961, Anagnostidis 1968). In the genus *Mastigocladus* numerous taxa were described, which do not belong to this genus (Komárek 2013). Among the *formae* described by Anagnostidis, some are subject to revision [e.g. M. fa. microchaetoides is "surely not *Mastigocladus*, as it is morphologically similar to *Aulosira*" according to Komárek (2013)]. The great morphological variability of this chroococcalean and nostocalean species should be further investigated in the future.

A number of species reported in Greece were found to be of not valid or of uncertain taxonomic status: The species *Pleurocapsa crepidinum* is possibly a *Chroococcidiopsis* species and needs revision (Komárek and Anagnostidis 2005). The species *Hydrocoleus homoeotrichus* *sensu* Anagnostidis et al. (1983), probably belongs to the Oscillatoriaceae and into the genus *Blennothrix* (Komárek and Anagnostidis 2005) according to its trichome structure. The taxon Aphanothece nidulans var. nidulans found in Lake Volvi (Hindak and Moustaka 1988) is not included either in the Chroococcales volume of Süsswasserflora von Mitteleuropa (Komárek and Anagnostidis 1999) nor in the AlgaeBase (Guiry and Guiry 2016); Algaebase contains only two valid *A. nidulans* varieties: var. longissima Nash and var. thermalis Hansgirg. *Gloeocapsa polydermatica* Kützing found in Nigrita hot springs (Anagnostidis 1961) is currently regarded as a taxonomic synonym of the chlorophyte *Sporotetras polydermatica* (Kützing) I.Kostikov, T.Darienko, A.Lukesová, & L.Hoffmann (Guiry and Guiry 2016). The taxon *Scolecia filosa* found in microbial bioerosion at an underwater marine site in Peloponnese (Färber et al. 2015) belongs to ichnotaxa produced by unknown heterotrophic organisms (Heindel et al. 2009), not cyanobacteria. The remaining 17 species belong to the family Pelonemataceae; this family, consisting of the genera *Achroonema*, *Pelonema*, *Peloploca*, and *Desmanthos*, was created to accommodate some colorless bacteria with multicellular filaments (trichomes), which were observed in hypolimnia of lakes and ponds or in sulfur springs (Hirsch 1981). At present, members of this family are considered unclassified below the Kingdom level (Guiry and Guiry 2016).

It is worth mentioning that 18 of the species included in the list have been initially described from Greek habitats. The marine epilithic *Ammatoidea aegea* described from Saronikos Gulf (Aegean Sea) (Anagnostidis and Pantazidou 1991a) is considered endemic to this area (Komarek and Anagnostidis 2005). Interestingly, most of these new species were found in rarely sampled habitats, such as caves [e.g. *Toxopsis calypsus*, *Ifinoe spelaea* (Lamprinou et al. 2011)], marine coastal rocks [e.g. epilithic *Cyanosarcina thalassia* (Anagnostidis and Pantazidou 1991b) or endolithic *Cyanosaccus aegaeus* and *Cyanosaccus atticus* (Anagnostidis and Pantazidou 1985, Anagnostidis and Pantazidou 1988c)], archaeological sites [e.g. aerophytic, chasmoendolithic *Cyanosarcina parthenonensis* (Anagnostidis and Pantazidou 1991b)], heliothermal saltwork mats [*Cyanothece halobia* (Anagnostidis and Roussomoustakaki 1991)], and waterfalls [*Phormidium edessae* (Skuja 1937)], highlighting the need for further research to reveal the unknown diversity in these environments. A recent study by Bravakos et al. (2016) on cyanobacteria strains isolated from mats of thermal springs of Greece indicates at least six new taxa at the genus level. Furthermore, Konstantinou et al. (2016) report possible new species on cyanobacteria strains isolated from marine sponges from the Aegean Sea, thus corroborating the abundant diversity of lesser-known environments. 

To the best of our knowledge, the list of Greek cyanobacteria compiled for the needs of the present study is the first list of both freshwater and marine Cyanophyceae in the Mediterranean. There is another list presenting 327 species from Israel (Tsarenko et al. 2000), but this refers only to the continental part of that country. The Egyptian list (Nassar 2014) contains 19 cyanobacteria species, whereas a phytoplankton checklist from the Eastern Adriatic (Vilicic et al. 2002) contains no cyanobacteria. Other local cyanobacterial lists were published in the past 15 years from continental parts of Europe, e.g. Slovakia, Sweden, Poland, Romania, Ukraine, Slovenia, the Netherlands, and the Czech Republic (Kaštovský et al. 2010 and references therein), as well as a checklist from the Baltic Sea reporting 172 cyanobacterial species (Hällfors 2004). The Czech Republic list comprises 505 species, 392 of which are considered recently present according to Kaštovský et al. 2010. The cyanobacteria checklist of Nepal (Rai et al. 2011) presents 274 species. Therefore, it appears that Greece hosts a comparatively high diversity of cyanobacteria, suggesting that the Mediterranean area is also a hot spot for the “substantially underestimated or unexplored” (Coll et al. 2010) microbes.

## Figures and Tables

**Figure 2a.**
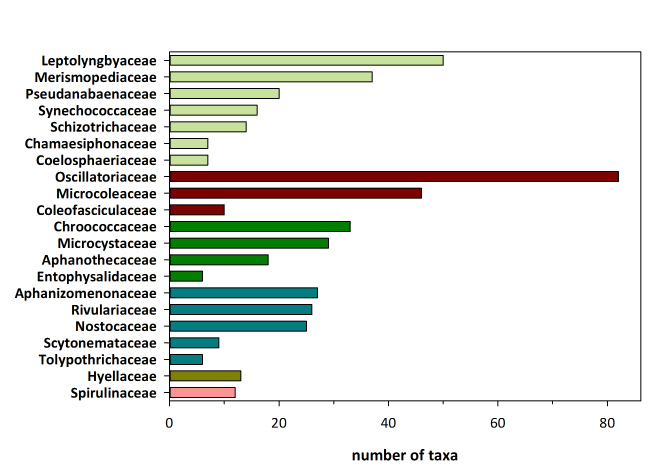


**Figure 2b.**
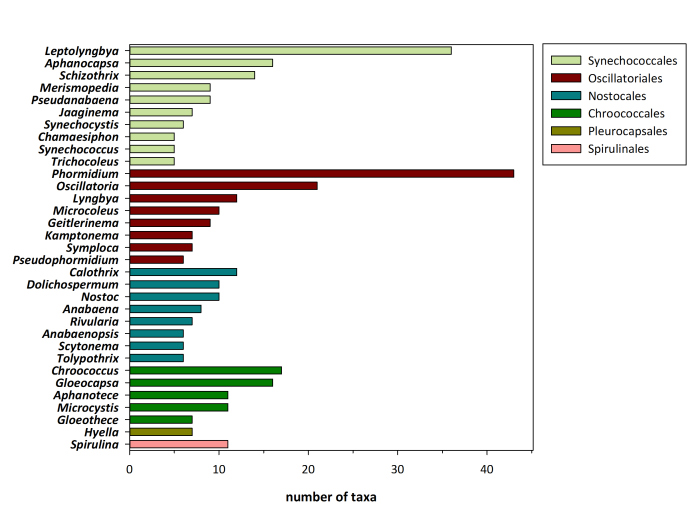


**Table 1.** List of infraspecific cyanobacterial taxa known to occur in Greece. For each taxon the first publication mentioning its occurrence in Greece is given.

**Species**	**Infraspecific epithet**	**Reference**
* Anabaena torulosa *	var. tenuis	Anagnostidis 1961
* Anabaena oscillarioides *	fa. circinalis	Anagnostidis 1968
* Aphanothece microscopica *	fa. endophytica	Anagnostidis 1968 ​
* Entophysalis deusta *	st. solentioides	Anagnostidis 1968 ​
	st. dermatocarpoides	Anagnostidis 1968 ​
	st. dermocarpoides	Anagnostidis 1968 ​
	st. gloeocapsoides	Anagnostidis 1968 ​
	st. hormathonematoides	Anagnostidis 1968 ​
	st. hyelloides	Anagnostidis 1968 ​
	st. pleurocapsoides	Anagnostidis 1968 ​
	st. tryponematoides	Anagnostidis 1968 ​
	st. typicus	Anagnostidis 1968 ​
* Gloeocapsa biformis *	st. derm. col. * ralfsianus *	Anagnostidis 1968 ​
	st. derm. * magma *	Anagnostidis 1968 ​
	st. dermochrous	Anagnostidis 1968 ​
	st. nannocytosus	Anagnostidis 1968 ​
	st. perdurans	Anagnostidis 1968 ​
	st. punctatus	Anagnostidis 1968 ​
* Gloeocapsa compacta *	st. perdurans	Anagnostidis 1968 ​
	st. lam. col. * magma*	Anagnostidis 1968 ​
	st. lam. col. * typicus *	Anagnostidis 1968 ​
	st. lam. * coloratus*	Anagnostidis 1968 ​
	st. nannocytosus	Anagnostidis 1968 ​
	st. perdurans	Anagnostidis 1968 ​
	st. simplex	Anagnostidis 1968 ​
* Gloeocapsa kützingiana*	st. lam. col. * magma*	Anagnostidis 1968 ​
	st. lam. * coloratus*	Anagnostidis 1968 ​
	st. perdurans	Anagnostidis 1968 ​
	st. rupestris	Anagnostidis 1968 ​
* Gloeocapsa sanguinea *	st. col. alp. * magma*	Anagnostidis 1968 ​
	st. col. alp. * ralfsianus*	Anagnostidis 1968 ​
	st. col. * ralfsianus*	Anagnostidis 1968 ​
	st. lam. col. alp. * magma*	Anagnostidis 1968 ​
	st. lam. col. * alpinus*	Anagnostidis 1968 ​
	st. lam. col. * magma*	Anagnostidis 1968 ​
	st. lam. * coloratus*	Anagnostidis 1968 ​
	st. perdurans	Anagnostidis 1968 ​
	st. simplex	Anagnostidis 1968 ​
* Mastigocladus laminosus *	fa. oscillariodes subfa. subrecta	Anagnostidis 1961
	fa. oscillariodes subfa. circinata	Anagnostidis 1961
	fa. oscillariodes subfa. spiroides	Anagnostidis 1961
	fa. pseudanabaenoides	Anagnostidis 1961
	fa. pseudanabaenoides subfa. subrecta	Anagnostidis 1961
	fa. pseudanabaenoides subfa. circinata	Anagnostidis 1961
	fa. pseudanabaenoides subfa. spiroides	Anagnostidis 1961
	fa. lyngbyoides	Anagnostidis 1961
	fa. lyngbyoides subfa. subrecta	Anagnostidis 1961
	fa. lyngbyoides subfa. circinata	Anagnostidis 1961
	fa. phormidioides	Anagnostidis 1961
	fa. phormidioides subfa. subrecta	Anagnostidis 1961
	fa. phormidioides subfa. circinata	Anagnostidis 1961
	fa. plectonematoides	Anagnostidis 1961
	fa. symplocoides	Anagnostidis 1961
	fa. anabaenoides	Anagnostidis 1961
	fa. anabaenoides subfa. subrecta	Anagnostidis 1961
	fa. anabaenoides subfa. circinata	Anagnostidis 1961
	fa. anabaenoides subfa. spiroides	Anagnostidis 1961
	fa. nostocoides	Anagnostidis 1961
	fa. aulosiroides	Anagnostidis 1961
	fa. stigonematoides	Anagnostidis 1961
	fa. microchaetoides	Anagnostidis 1961
	fa. tolypotrichoides	Anagnostidis 1961
	fa. scytonematoides	Anagnostidis 1961
	fa. typica	Anagnostidis 1961
	fa. typica subfa. normalis	Anagnostidis 1961
	fa. typica subfa. brachytrichoides	Anagnostidis 1961
	fa. pallidus	Anagnostidis 1961
	fa. oscillariodes	Anagnostidis 1961
	fa. chroococcoides	Anagnostidis 1961
	fa. pleurocapsoides	Anagnostidis 1961
* Nostoc punctiforme *	var. populorum ^1^	Anagnostidis and Economou-Amilli 1978
* Pseudanabaena articulata *	fa. ^2^	Anagnostidis 1968 ​
* Pseudanabaena lonchoides *	fa. circinata	Anagnostidis 1961
	fa. crassior	Anagnostidis 1968 ​
	fa. tenuis	Anagnostidis 1968 ​
* Scytonema myochrous *	st. crustaceus	Anagnostidis 1968 ​
	st. petalonema	Anagnostidis 1968 ​
	st. petalonema	Anagnostidis 1968 ​
	st. petalonema	Anagnostidis 1968 ​
	st. typicus	Anagnostidis 1968 ​
* Siphononema polonicum *	st. chamaesiphonoides	Anagnostidis 1968 ​
	st. juvenilis	Anagnostidis 1968 ​
	st. scopulonematoides	Anagnostidis 1968 ​
	st. stigonematoides	Anagnostidis 1968 ​
* Synechocystis aquatilis *	var. minor	Anagnostidis 1961

^1^ The current taxonomic status is Nostoc punctiforme fa. populorum

^2^ The author does not identify a certain forma

**Table 2.** List of cyanobacterial taxa known to occur in Greece with uncertain or not valid taxonomic status. For each taxon the publication mentioning its occurrence in Greece and the current taxonomic status is given.

**Taxon**	**Reference**	**Current taxonomic status**
*Achroonema lentum*	Anagnostidis and Economou–Amilli 1980	unclassified below the Kingdom level (Guiry and Guiry 2016)
*Achroonema proteiforme*	Anagnostidis 1968	
*Achroonema angustum*	Anagnostidis 1968	
*Achroonema atriculatum*	Anagnostidis 1968	
*Achroonema splendens*	Anagnostidis 1968	
*Achroonema subsalsum*	Anagnostidis 1968	
*Desmanthos thiocrenophilum (?)*	Anagnostidis 1968	
*Pelonema aphane*	Anagnostidis 1968	
Pelonema cf. subtilissimum	Anagnostidis and Roussomoustakaki 1991	
*Pelonema pseudovacuolatum*	Anagnostidis 1968	
*Pelonema subtilissimum*	Anagnostidis 1968	
*Pelonema tenue*	Anagnostidis 1968	
*Peloploca ferruginea (?)*	Anagnostidis 1968	
*Peloploca taeniata*	Anagnostidis 1968	
*Peloploca undulata*	Anagnostidis 1968	
*Pleurocapsa crepidinum*	Anagnostidis and Golubic 1966​	needs revision; possibly a *Chroococcidiopsis* species (Komárek and Anagnostidis 2005​)
Aphanothece nidulans var. nidulans	Hindak and Moustaka 1988	uncertain; Algabase (Guiry and Guiry 2016) contains only two valid *A. nidulans* varieties: var. longissima Nash and var. thermalis Hansgirg
*Hydrocoleus homoeotrichus*	Anagnostidis et al. 1983	needs revision; *sensu* Anagnostidis et al 1983, probably belongs to the Oscillatoriaceae according to trichome structure, and into the genus *Blennothrix* (Komárek and Anagnostidis 2005)
*Achroonema pseudangustum*	Anagnostidis and Economou–Amilli 1980	not valid (Guiry and Guiry 2016)
*Scolecia filosa*	Färber et al. 2015	Ichnotaxa produced by unknown heterotrophic organisms (Heindel et al. 2009)
*Gloeocapsa polydermatica*	Anagnostidis 1961	*Sporotetras polydermatica* [Chlorophyta] (Guiry and Guiry 2016​)

## References

[B3352624] Anagnostidis K (1961). Investigations of the Cyanophyceae in some thermal springs in Greece.

[B3352633] Anagnostidis K (1968). Investigations of Sulphur-communities (Sulphuretum) in marine and freshwater habitats. A taxonomic, floristic, ecological, phytosociological, phytogeographic study.

[B3352754] Anagnostidis K. (1977). Some remarks on the taxonomy of the genus *Borzia* Cohn ex Gom. from Greece. Schweizerische Zeitschrift für Hydrologie.

[B3352764] Anagnostidis K., Economou-Amilli A. (1978). Microorganisms from the volcano of Nea Kammeni Island (Santorini). Thera and the Aegean World.

[B3362075] Anagnostidis K, Economou–Amilli A (1980). Limnological studies on Lake Pamvotis (Ioannina), Greece I. Hydroclimatology, phytoplankton - periphyton with special reference to the valency of some microorganisms from sulphureta as bioindicators. Archiv für Hydrobiologie.

[B3352774] Anagnostidis K., Golubic S. (1966). Über die Okologie einiger *Spirulina* Arten. Nova Hedwigia.

[B3352784] Anagnostidis K, Komárek J (1985). Modern approach to the classification system of cyanophytes. 1- Introduction. Arch. Hydrobiol. Suppl..

[B3352794] Anagnostidis K, Komárek J (1988). Modern approach to the classification system of cyanophytes. 3- Oscillatoriales.. Archiv für Hydrobiologie, Supplement.

[B3352804] Anagnostidis K, Komárek J (1990). Modern approach to the classification system of cyanophytes. 5- Stigonematales.. Archiv für Hydrobiologie, Supplement.

[B3352814] Anagnostidis K., Pantazidou A. (1985). *Cyanosaccus
aegaeus* n. sp., a new marine endolithic cyanophyte from the Aegean Sea, Hellas (Greece). Archiv für Hydrobiologie (Algological Studies).

[B3352844] Anagnostidis K., Pantazidou A. (1988). Endolithic cyanophytes from the saline thermal springs of Aedipsos, Hellas (Greece). Algological Studies/Archiv für Hydrobiologie, Supplement.

[B3352834] Anagnostidis K., Pantazidou A. (1988). *Hyella
kalligrammos* sp. nov., *Hyella
maxima* (Geitl.) comb. nov., and other freshwater morphotypes of the genus *Hyella* Born. et Flah. (Chroococcales, Cyanophyceae). Algological Studies/Archiv für Hydrobiologie, Supplement.

[B3352824] Anagnostidis K., Pantazidou A. (1988). *Cyanosaccus
atticus*, a new marine euendolithic chroococcoid cyanophyte in relation to the epilithic Podocapsa Erceg.. Algological Studies/Archiv für Hydrobiologie, Supplement.

[B3352864] Anagnostidis K., Pantazidou A. (1991). *Ammatoidea
aegaea* (Oscillatoriales), a new marine epilithic species from the Aegean Sea, Hellas, with a reference to the validity of the genus *Ammatoidea*. Algological Studies/Archiv für Hydrobiologie, Supplement.

[B3352854] Anagnostidis K., Pantazidou A. (1991). Marine and aerophytic *Cyanosarcina*, *Stanieria* and *Pseudocapsa* (Chroococcales) species from Hellas (Greece). Algological Studies/Archiv für Hydrobiologie, Supplement.

[B3352874] Anagnostidis K., Roussomoustakaki M. (1988). Cyanophytes from metal burdened substrates. Algological Studies/Archiv für Hydrobiologie, Supplement.

[B3352884] Anagnostidis K., Roussomoustakaki M. (1991). *Isocystis
halobia* spec. nova, a benthic nostocalean cyanophyte from the heliothermal saltwork mats of Messolongion, Hellas (Greece). Algological Studies.

[B3352894] Anagnostidis K., Economou-Amilli A., Makris K. (1988). On the morphotypes of *Phormidium
boryanum* (Bory ex Gom.) Anagn. et Kom. and *Phormidium
janthiphorum* (Fiori-Mazz. ex Gom.) Elenk. A taxonomic consideration. Algological Studies/Archiv für Hydrobiologie, Supplement.

[B3352904] Anagnostidis K., Economou-Amilli A., Overbeck J. (1988). *Anabaena
azollae* Strasb. and the periphyton of *Azolla
filiculoides* Lam. in lake Trichonis and the lagoon of Aetolikon, Hellas (Greece). Archiv für Hydrobiologie..

[B3352914] Anagnostidis K., Economou-Amilli A., Pantazidou A. (1982). Studies on microflora of Perama Cave, Ioannina, Greece. Bulletin de la Société Spéléologique de Grèce.

[B3352924] Anagnostidis K., Economou-Amilli A., Roussomoustakaki M. (1983). Epilithic and chasmolithic microflora (Cyanophyta, Bacillariophyta) from marbles of the Parthenon-Acropolis, Athens, Greece. Nova Hedwigia.

[B3352934] Anagnostidis K., Economou-Amilli A., Tsangridis A. (1981). Taxonomic and floristic studies of algae from rice-fields of Kalochorion-Thessaloniki, Greece. Nova Hedwigia.

[B3352944] Ananiadis C. I. (1956). Limnological study of Lake Karla. Bulletin de L'Institut Océanographique.

[B3352954] Bailly N., Gerovasileiou V., Arvanitidis C., Legakis A. (2016). Introduction to the Greek Taxon Information System (GTIS) in LifeWatchGreece: the construction of the Preliminary Checklists of Species of Greece.. Biodiversity Data Journal.

[B3352596] Batler Samuel (1998). Homer's Iliad.

[B3352642] Bravakos Panos, Kotoulas Georgios, Skaraki Katerina, Pantazidou Adriani, Economou-Amilli Athena (2016). A polyphasic taxonomic approach in isolated strains of Cyanobacteria from thermal springs of Greece. Molecular Phylogenetics and Evolution.

[B3365856] Christodoulou Maria, Meletiou-Christou Maria-Sonia, Parmakelis Aristeidis, Economou-Amilli Athena, Pantazidou Adriani (2015). Further findings from Daveli Cave (Attica, Greece) enhancing the establishment of the genus*Oculatella* (Pseudanabaenaceae, Cyanobacteria).. Phytotaxa.

[B3363341] Coll Marta, Piroddi Chiara, Steenbeek Jeroen, Kaschner Kristin, Rais Lasram Frida Ben, Aguzzi Jacopo, Ballesteros Enric, Bianchi Carlo Nike, Corbera Jordi, Dailianis Thanos, Danovaro Roberto, Estrada Marta, Froglia Carlo, Galil Bella S., Gasol Josep M., Gertwagen Ruthy, Gil João, Guilhaumon François, Kesner-Reyes Kathleen, Kitsos Miltiadis-Spyridon, Koukouras Athanasios, Lampadariou Nikolaos, Laxamana Elijah, la Cuadra Carlos M. López-Fé de, Lotze Heike K., Martin Daniel, Mouillot David, Oro Daniel, Raicevich Saša, Rius-Barile Josephine, Saiz-Salinas Jose Ignacio, Vicente Carles San, Somot Samuel, Templado José, Turon Xavier, Vafidis Dimitris, Villanueva Roger, Voultsiadou Eleni (2010). The Biodiversity of the Mediterranean Sea: Estimates, Patterns, and Threats. PLoS ONE.

[B3363419] Danielidis D. B., Spartinou M., Economou-Amilli A. (1996). Limnological survey of Lake Amvrakia, western Greece. Hydrobiologia.

[B3352653] Duval B, Duval E, Hoham R W (1999). Snow algae of the Sierra Nevada, Spain, and High Atlas mountains of Morocco.. International microbiology : the official journal of the Spanish Society for Microbiology.

[B3363405] Economou-Amilli A., Anagnostidis K., Roussomoustakaki M. (1984). Structural Aspects of the Adaptation of Some Blue-Green Algae and Diatoms to Desiccation. Tasks for vegetation science.

[B3362095] Färber Claudia, Wisshak Max, Pyko Ines, Bellou Nikoleta, Freiwald André (2015). Effects of Water Depth, Seasonal Exposure, and Substrate Orientation on Microbial Bioerosion in the Ionian Sea (Eastern Mediterranean). PLOS ONE.

[B3362574] Gkelis Spyros, Panou Manthos (2016). Capturing biodiversity: linking a cyanobacteria culture collection to the "scratchpads" virtual research environment enhances biodiversity knowledge.. Biodiversity data journal.

[B3362607] Gkelis Spyros, Zaoutsos Nikos (2014). Cyanotoxin occurrence and potentially toxin producing cyanobacteria in freshwaters of Greece: A multi-disciplinary approach. Toxicon.

[B3362530] Gkelis Spyros, Lanaras Thomas, Sivonen Kaarina (2015). Cyanobacterial Toxic and Bioactive Peptides in Freshwater Bodies of Greece: Concentrations, Occurrence Patterns, and Implications for Human Health. Marine Drugs.

[B3362540] Gkelis Spyros, Tussy Pablo Fernández, Zaoutsos Nikos (2015). Isolation and preliminary characterization of cyanobacteria strains from freshwaters of Greece. Open Life Sciences.

[B3363395] Gkelis S., Moustaka-Gouni M., Sivonen K., Lanaras T. (2005). First report of the cyanobacterium Aphanizomenon
ovalisporum Forti in two Greek lakes and cyanotoxin occurrence. Journal of Plankton Research.

[B3362617] Gkelis Spyros, Papadimitriou Theodoti, Zaoutsos Nikos, Leonardos Ioannis (2014). Anthropogenic and climate-induced change favors toxic cyanobacteria blooms: Evidence from monitoring a highly eutrophic, urban Mediterranean lake. Harmful Algae.

[B3362550] Gkelis S., Rajaniemi P., Vardaka E., Moustaka-Gouni M., Lanaras T., Sivonen K. (2005). *Limnothrix
redekei* (Van Goor) Meffert (Cyanobacteria) Strains from Lake Kastoria, Greece Form a Separate Phylogenetic Group. Microbial Ecology.

[B3352745] Guiry M. D., Guiry M. D. AlgaeBase. World-wide electronic publication, National University of Ireland, Galway.. http://www.algaebase.org.

[B3362066] Hällfors Guy (2004). Checklist of Baltic Sea Phytoplankton Species.

[B3362106] Heindel K., Westphal H., Wisshak M. (2009). Data report: bioerosion in the reef framework, IODP Expedition 310 off Tahiti (Tiarei, Maraa, and Faaa sites).

[B3362085] Hindak F., Moustaka M. (1988). Planktic cyanophytes of Lake Volvi, Greece. Algological Studies/Archiv für Hydrobiologie, Supplement Volumes.

[B3365846] Hindák F. (1988). Taxonomic position of two cyanophyte genera, *Cyanostylon* Geitler and *Siphonosphaera* gen. nov.. Archiv für Hydrobiologie/Algological Studies.

[B3365836] Hindák F. (1993). To the taxonomy of the chroococcal genus *Pannus* Hickel 1991 (Cyanophyta/Cyanobacteria). Archiv für Hydrobiologie/Algological Studies.

[B3352445] Hirsch Peter (1981). The Family Pelonemataceae.. The Prokaryotes.

[B3352566] Kaštovský Jan, Hauer Tomas, Komarek Jiri, Skacelova Olga (2010). The list of cyanobacterial species of the Czech Republic to the end of 2009.. Fottea.

[B3362637] Katsiapi Matina, Mazaris Antonios D., Charalampous Evangelia, Moustaka-Gouni Maria (2012). Watershed land use types as drivers of freshwater phytoplankton structure. Hydrobiologia.

[B3362627] Katsiapi Matina, Moustaka-Gouni Maria, Michaloudi Evangelia, Kormas Konstantinos Ar. (2011). Phytoplankton and water quality in a Mediterranean drinking-water reservoir (Marathonas Reservoir, Greece). Environmental Monitoring and Assessment.

[B3362154] Komárek Jiří (2013). Phenotypic characters of heterocytous cyanobacteria.. Süßwasserflora von Mitteleuropa, Bd. 19/3: Cyanoprokaryota.

[B3363430] Komárek Jiří (2016). Review of the cyanobacterial genera implying planktic species after recent taxonomic revisions according to polyphasic methods: state as of 2014. Hydrobiologia.

[B3362115] Komárek J, Anagnostidis K (1986). Modern approach to the classification system of cyanophytes. 2- Chroococcales.. Archiv für Hydrobiologie, Supplement Volumes.

[B3362125] Komárek J, Anagnostidis K (1989). Modern approach to the classification system of cyanophytes. 4- Nostocales.. Archiv für Hydrobiologie, Supplement.

[B3362511] Komárek J., Anagnostidis K. (1999). Cyanoprokaryota 1. Teil: Chroococcales..

[B3362145] Komárek J, Anagnostidis K (2005). Cyanoprokaryota 2. Teil/ 2nd Part: Oscillatoriales..

[B3363440] Komárek J, Kaštovský J., Mareš J., Johansen J. R. (2014). Taxonomic classification of cyanoprokaryotes (cyanobacterial genera) 2014, using a polyphasic approach. Preslia.

[B3363832] Konstantinou Despoina, Gerovasileiou Vasilis, Voultsiadou Eleni, Gkelis Spyros (2016). 41st CIESM Congress Proceedings.

[B3362647] Koureas Dimitrios, Hardisty Alex, Vos Rutger, Agosti Donat, Arvanitidis Christos, Bogatencov Peter, Buttigieg Pier Luigi, Jong Yde de, Horvath Ferenc, Gkoutos Georgios, Groom Quentin, Kliment Tomas, Kõljalg Urmas, Manakos Ioannis, Marcer Arnald, Marhold Karol, Morse David, Mergen Patricia, Penev Lyubomir, Pettersson Lars, Svenning Jens-Christian, de Putte Anton van, Smith Vincent (2016). Unifying European Biodiversity Informatics (BioUnify). Research Ideas and Outcomes.

[B3362168] Lamprinou Vasiliki, Economou-Amilli Athena, Danielidis Daniel, Pantazidou Adriani (2012). Distribution survey of Cyanobacteria in three Greek caves of Peloponnese. International Journal of Speleology.

[B3362178] Lamprinou Vasiliki, Danielidis Daniel, Pantazidou Adriani, Oikonomou Alexandra, Economou-Amilli Athena (2014). The show cave of Diros vs. wild caves of Peloponnese, Greece - distribution patterns of Cyanobacteria.. International Journal of Speleology.

[B3362189] Lamprinou Vasiliki, Pantazidou Adriani, Papadogiannaki Georgia, Radea Canella, Economou-Amilli Athena (2009). Cyanobacteria and associated invertebrates in Leontari Cave.. Fottea.

[B3362200] Lamprinou V., Hernandez-Marine M., Canals T., Kormas K., Economou-Amilli A., Pantazidou A. (2011). Morphology and molecular evaluation of *Iphinoe
spelaeobios* gen. nov., sp. nov. and *Loriellopsis
cavernicola* gen. nov., sp. nov., two stigonematalean cyanobacteria from Greek and Spanish caves. INTERNATIONAL JOURNAL OF SYSTEMATIC AND EVOLUTIONARY MICROBIOLOGY.

[B3362562] Lamprinou Vasiliki, Hernandez-Marine Mariona, Pachiadaki Maria G., Kormas Konstantinos A., Economou-Amilli Athena, Pantazidou Adriani (2013). New findings on the true-branched monotypic genus Iphinoe (Cyanobacteria) from geographically isolated caves (Greece).. Fottea.

[B3362212] Lamprinou V., Skaraki K., Kotoulas G., Anagnostidis K., Economou-Amilli A., Pantazidou A. (2013). A new species of *Phormidium* (Cyanobacteria, Oscillatoriales) from three Greek Caves: morphological and molecular analysis. Fundamental and Applied Limnology / Archiv für Hydrobiologie.

[B3362224] Lanaras T., Tsitsamis S., Chlichlia C., Cook C. M. (1989). Toxic cyanobacteria in Greek freshwaters. Journal of Applied Phycology.

[B3363385] Metaxatos Angelina, Panagiotopoulos Christos, Ignatiades Lydia (2003). Monosaccharide and aminoacid composition of mucilage material produced from a mixture of four phytoplanktonic taxa. Journal of Experimental Marine Biology and Ecology.

[B3362234] Montesanto B, Ziller S, Danielidis D, Economou-Amilli A (1999). Phytoplankton community structure in the lower reaches of a Mediterranean river (Aliakmon, Greece). Fundamental and Applied Limnology.

[B3362244] Moustaka-Gouni M. (1988). The structure and dynamics of the phytoplankton assemblages in Lake Volvi, Greece I. Phytoplankton composition and abundance during the period March 1984-March 1985.. Archiv für Hydrobiologie.

[B3362254] Moustaka-Gouni Maria (1993). Phytoplankton succession and diversity in a warm monomictic, relatively shallow lake: Lake Volvi, Macedonia, Greece. Intermediate Disturbance Hypothesis in Phytoplankton Ecology.

[B3362268] Moustaka-Gouni M., Nikolaidis G. (1990). Phytoplankton of a warm monomictic lake - Lake Vegoritis, Greece.. Archiv für Hydrobiologie.

[B3362520] Moustaka-Gouni M., Nikolaidis G. (1992). Phytoplankton and physical-chemical features of Tavropos Reservoir, Greece. Hydrobiologia.

[B3362278] Moustaka-Gouni M, Albanakis K., Mitrakas M., Psilovikos A. (2000). Planktic autotrophs and environmental conditions in the newly-formed hydroelectric Thesaurus reservoir, Greece. Fundamental and Applied Limnology.

[B3362288] Moustaka-Gouni M., Michaloudi E., Katsiapi M., Genitsaris S. (2007). Greece : The coincidence of an *Arthrospira* -*Anabaenopsis* bloom and the mass mortality of birds in Lake Koronia. Harmful algae news.

[B3362584] Moustaka-Gouni Maria, Kormas Konstantinos Ar., Vardaka Elisabeth, Katsiapi Matina, Gkelis Spyros (2009). *Raphidiopsis
mediterranea* Skuja represents non-heterocytous life-cycle stages of *Cylindrospermopsis
raciborskii* (Woloszynska) Seenayya et Subba Raju in Lake Kastoria (Greece), its type locality: Evidence by morphological and phylogenetic analysis. Harmful Algae.

[B3362595] Moustaka-Gouni M., Kormas K. A., Polykarpou P., Gkelis S., Bobori D. C., Vardaka E. (2010). Polyphasic evaluation of *Aphanizomenon
issatschenkoi* and *Raphidiopsis
mediterranea* in a Mediterranean lake. Journal of Plankton Research.

[B3362298] Moustaka-Gouni Maria, Hiskia Anastasia, Genitsaris Savvas, Katsiapi Matina, Manolidi Korina, Zervou Sevasti-Kiriaki, Christophoridis Christophoros, Triantis Theodoros M., Kaloudis Triantafyllos, Orfanidis Sotiris (2016). First report of *Aphanizomenon
favaloroi* occurrence in Europe associated with saxitoxins and a massive fish kill in Lake Vistonis, Greece. Marine and Freshwater Research.

[B3362324] Moustaka M. (1988). SEASONAL VARIATIONS, ANNUAL PERIODICITY AND SPATIAL DISTRIBUTION OF PHYTOPLANKTON IN LAKE VOLVI.

[B3362031] Nassar Mohamed (2014). Checklist of Phytoplankton Species in the Egyptian Waters of the Red Sea and Some Surrounding Habitats (1990-2010). Annual Research & Review in Biology.

[B3362344] Overbeck J., Anagnostidis K., Economou-Amilli A. (1982). A limnological survey of three Greek lakes, Trichonis, Lyssimachia and Amvrakia. Archiv fur Hydrobiologie.

[B3362354] Pantazidou A., Louvrou I., Economou-Amilli A. (2006). Euendolithic shell-boring cyanobacteria and chlorophytes from the saline lagoon Ahivadolimni on Milos Island, Greece. European Journal of Phycology.

[B3362364] Papadimitriou Th., Katsiapi M., Kormas K. Ar., Moustaka-Gouni M., Kagalou I. (2013). Artificially-born “killer” lake: Phytoplankton based water quality and microcystin affected fish in a reconstructed lake. Science of The Total Environment.

[B3352605] Peck A. L. (1970). Aristotle. History of Animals. Books IV-VI. English translation, Introduction and Comments..

[B3362375] Radea Canella, Louvrou Ioanna, Pantazidou Adriani, Amilli Athena Economou- (2010). Photosynthetic microorganisms as epibionts and euendoliths on biotic substrates in a thermal spring with ferric-iron deposits.. Fottea.

[B3362021] Rai S. K., Rai R. K., Jha S. (2011). Cyanobacteria of Nepal: A Checklist with Distribution. Our Nature.

[B3352586] Skuja H. (1937). Süsswasseralgen aus Griechenland und Kleinasien. Hedwigia.

[B3352576] Stanković S. (1931). Sur les particularités limnologiques de lacs égéens.. Verhandlungen des Internationalen Verein Limnologie.

[B3362385] Tafas T., Economou-Amilli A. (1997). Limnological survey of the warm monomictic lake Trichonis (central western Greece)*II. Seasonal phytoplankton periodicity – a community approach*. Hydrobiologia.

[B3362395] Temponeras M., Kristiansen J., Moustaka-Gouni M. (2000). Seasonal variation in phytoplankton composition and physical-chemical features of the shallow Lake Doïrani, Macedonia, Greece. The Trophic Spectrum Revisited.

[B3362409] Tryfon E. (1996). Pannus
spumosus (Chroococcales, Cyanoprocaryota) from Lake Mikri Prespa, Greece. Phycologia.

[B3362419] Tryfon Eleni, Moustaka-Gouni Maria, Nikolaidis Georgios (1996). Phytoplankton and Nutrients in the River Strymon, Greece. Internationale Revue der gesamten Hydrobiologie und Hydrographie.

[B3362429] Tryfon E., Moustaka-Gouni M., Nikolaidis G. (1997). Planktic cyanophytes and their ecology in the shallow Lake Mikri Prespa, Greece. Nordic Journal of Botany.

[B3362000] Tsarenko P. M., Vinogradova O. N., Stupina V. V., Wasser Solomon P., Nevo Eviatar (2000). Diversity of algae in the continental part of Israel. International Journal on Algae.

[B3362439] Vardaka E., Moustaka-Gouni M., Lanaras T. (2000). Temporal and spatial distribution of planktic cyanobacteria in Lake Kastoria, Greece, a shallow, urban lake. Nordic Journal of Botany.

[B3362459] Vardaka Elizabeth, Moustaka-Gouni Maria, Cook Catherine M., Lanaras Tom (2005). Cyanobacterial blooms and water quality in Greek waterbodies. Journal of Applied Phycology.

[B3362469] Vareli Katerina, Briasoulis Evangelos, Pilidis George, Sainis Ioannis (2009). Molecular confirmation of *Planktothrix
rubescens* as the cause of intense, microcystin—Synthesizing cyanobacterial bloom in Lake Ziros, Greece. Harmful Algae.

[B3362041] Vilicic D., Marasovic I., Miokovic D. (2002). Checklist of phytoplankton in the eastern Adriatic Sea. Acta Bot. Croat..

[B3362479] Voultsiadou Eleni (2007). Sponges: an historical survey of their knowledge in Greek antiquity. Journal of the Marine Biological Association of the UK.

